# The Application of EM38: Determination of Soil Parameters, Selection of Soil Sampling Points and Use in Agriculture and Archaeology

**DOI:** 10.3390/s17112540

**Published:** 2017-11-04

**Authors:** Kurt Heil, Urs Schmidhalter

**Affiliations:** Chair of Plant Nutrition, Technical University of Munich, Emil-Ramann-Str. 2, D-85350 Freising, Germany; schmidhalter@wzw.tum.de

**Keywords:** EM38, apparent electrical conductivity, soil mapping, yield variability and management zones, soil sampling schemes, soil types

## Abstract

Fast and accurate assessment of within-field variation is essential for detecting field-wide heterogeneity and contributing to improvements in the management of agricultural lands. The goal of this paper is to provide an overview of field scale characterization by electromagnetic induction, firstly with a focus on the applications of EM38 to salinity, soil texture, water content and soil water turnover, soil types and boundaries, nutrients and N-turnover and soil sampling designs. Furthermore, results concerning special applications in agriculture, horticulture and archaeology are included. In addition to these investigations, this survey also presents a wide range of practical methods for use. Secondly, the effectiveness of conductivity readings for a specific target in a specific locality is determined by the intensity at which soil factors influence these values in relationship to the desired information. The interpretation and utility of apparent electrical conductivity (EC_a_) readings are highly location- and soil-specific, so soil properties influencing the measurement of EC_a_ must be clearly understood. From the various calibration results, it appears that regression constants for the relationships between EC_a_, electrical conductivity of aqueous soil extracts (EC_e_), texture, yield, etc., are not necessarily transferable from one region to another. The modelling of EC_a_, soil properties, climate and yield are important for identifying the location to which specific utilizations of EC_a_ technology (e.g., EC_a−_texture relationships) can be appropriately applied. In general, the determination of absolute levels of EC_a_ is frequently not possible, but it appears to be quite a robust method to detect relative differences, both spatially and temporally. Often, the use of EC_a_ is restricted to its application as a covariate or the use of the readings in a relative sense rather than as absolute terms.

## 1. Introduction

Fast and accurate detection of within-field variation is essential for the detection and management of the environment. The EM38 device (Geonics. Ltd., Mississauga, ON, Canada), a sensor that delivers dense datasets, can be used to accomplish this goal. The EM38 meter is the most widely used EMI sensor in agriculture [1,2].

Researchers have related EM38-EC_a_ (apparent electrical conductivity—EC_a_) to a number of different soil properties either within an individual field or across the entire landscape [3]. The application of EM38 began with the detection of salinity and continued with the determination of clay and water content [2]. Currently, areas of application include the estimation of nutrient levels and other soil chemical and physical properties, soil sampling points, the determination of soil types and their boundaries, the prediction of yield and the delineation of crop management zones. The increasing application especially during the last decade is also caused by various technical developments: Global Positioning Systems (GPS), surface mapping programs and systems for data analysis and interpretation. Technical data, construction and tool specification are described in Heil and Schmidhalter [4].

**Figure 1:**
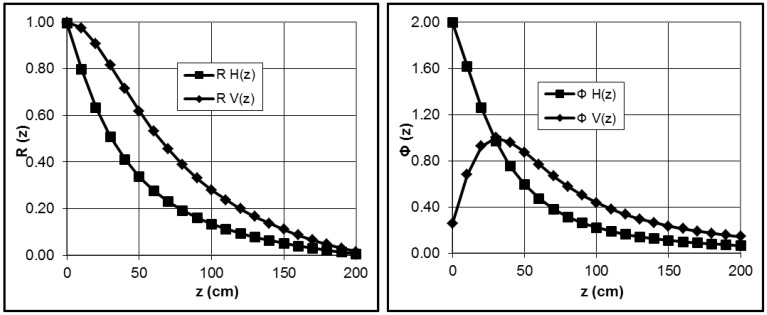
(**Left**) Relative cumulative contribution vs depth for vertically (RV(*z*)) and horizontally (RH(*z*)) orientated dipoles; (**Right**) Comparison of the relative responses for vertically (FV(*z*)) and horizontally (FH(*z*)) oriented dipoles.

This device consists of a receiver and a transmitter coil installed 1.0 m apart at the opposite ends of a nonconductive bar. The investigated depth range depends on the coil configuration and the distance between the coils. While the distance is fixed, the orientation of the coils can be changed. In the vertical mode, the device is in a position perpendicular to the soil, whereas in the second case, the device lies parallel to the soil surface [5,6]. The sensitivity in the horizontal mode is the highest directly below the instrument, while the sensitivity in the vertical position reaches a maximum at approximately 30–40 cm below the instrument. The depth-weighted nonlinearity of the response is shown in Figure 1. The cumulative relative contributions of all soil EC are R(z).

An exact determination of the measurement depth is difficult. Theoretically, the readings acquire an unlimited depth, but in reality, it depends on the electrical contrast. The most common definition is a depth range up to 1.5 m when using the vertical dipole mode and 0.75 m in the case of the horizontal mode [4,5,6].

**Figure 2:**
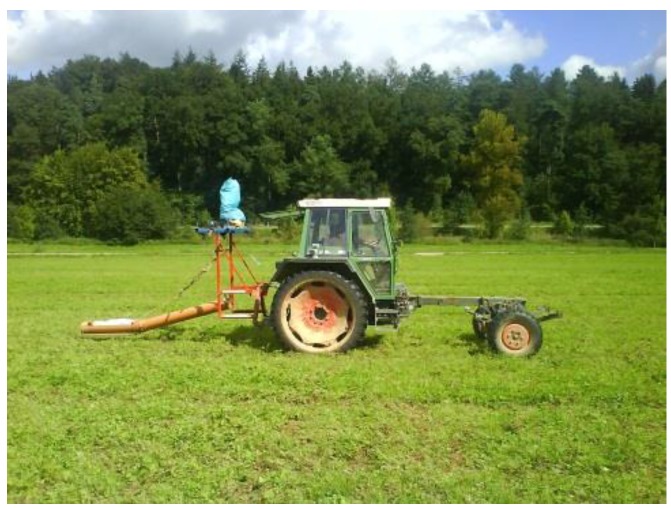
Mounting of the EM38 on a metal-free sledge pulled by a tractor (constructed after Corwin and Lesch [7]).

For wide area measurements e.g., in precision agriculture as well as in field-scale soil property measurements the sensor is mounted on metal-free sledge and pulled behind an all-terrain vehicle equipped with a GPS receiver and data collection computer (Figure 2).

Beside the EM38, EM31 and EM34 electromagnetic devices are also available on the market. In contrast to the EM38, the other devices are designed for the detection of deeper areas of soils, e.g., geological layers, ground water and other subsurface feature associated with changes in ground conductivity. The EM31 has an effective exploration depth of about six metres with an intercoil spacing of 3.66 m. The EM34-3 uses three intercoil spacings—10, 20 and 40 m—to provide variable depths of exploration down to 60 m.

## 2. Goal of this Study

The objective of this study is to summarize the results of recent measurements and the development of algorithms from EC_a_ measurements obtained with the geophysical sensor EM38. Given the numerous possible subject matters for research in using EM38, this review paper has focused on the following specific fields:

1. Salinity
2. Soil-related properties in non-saline soils
  - Soil texture
  - Soil water content, water balance
  - Soil horizons and vertical discontinuities
  - N-turnover, cation exchange capacity, organic matter and additional soil parameters
  - Soil sampling designs
  - Soil type boundaries
3. Agriculture
  - Agricultural yield variability and management zones
  - Efficiency of agricultural field experimentation
  - Additional application of EM38 in agriculture and horticulture
4. Archaeology

The rationale of this compilation should allow the users of this sensor to understand which variables are today detectable, which objectives are realistic and in which regions applications are widely used. The users have to note that these sensor readings are a composite of soil properties and therefore not a replacement for in-depth knowledge’s about soil and site.

## 3. Surveying Soil Salinity

Ample information can be found in the literature that describes the potential of EM-38 measurements for the non-invasive detection of in situ soil salinity (Table 1).

Corwin and Lesch [97] summarized five methods that have been used for determining soil salinity in the field: (1) visual crop observations; (2) the electrical conductivity of the soil solution (soil paste or extracts); (3) in situ measurement of electrical conductivity with electrical resistivity (with the Wenner array method); (4) non-invasive measurement of electrical conductivity with EC_a_ and, most recently; (5) in situ measurement of electrical conductivity with time domain reflectometry. Frequently, EC_e_ (e.g., conductivity of aqueous extracts of soil saturated soil paste, EC_1:5_, EC_1:2_ or EC_1:1,_ conductivity of soil: water suspensions) was indicated as the most useful and reliable measurement of point-wise salinity detection [43,44,45,46,47,48,49,50,51,52,53,54,55,56,57,81,82,95,96,97,98]. In older publications, EC_e_ alone was often used to identify salt-affected areas [29,30,31,32,33,34,35,36,37,38,39,40,41,42,43,44,45,46,47,48,49,50,51,52,53,54,55,56,57,81,82,86,93,95,96,97,98,99] Norman [30] developed a salinity classification system based on the range of total dissolved salt concentration (EC_1:5_) with corresponding groupings of crops with different tolerances to root zone salinity. Soil salinity can be derived from the conductivity of the bulk soil (EC_a_). For example, salinity is quantified and monitored in irrigated agricultural areas of arid zones by means of EC_a_ measurements using EM38 [28,29,30,31,32,33,34,35,36,37,38,39,40,86]. In areas where saline soils exist, 65% to 70% of the variance in EC_a_ can be explained by the changes in salinity alone [51]. EC_a_ readings can be used to predict the exchangeable sodium percentage and EC_e_ as well [27]. The different terms of salinity can be inferred from the equation EC_a_ = *f*(EC_e(0−*Z* cm)_).

During the last three decades, several calibration methods have been published describing EM38-EC_e_ relationships [27,28,41,60]. Following the classification of Triantafilis et al. [43] and Vlotman et al. [49], further calibration approaches have been proposed, using linear regression, multiple regression coefficients [15,31,37], simple depth weighted coefficients [5,13,14,15,16,17,18,19,20,21,22,23,24,25,26,27,28,29,30,31,32,33,34,35,36,37,38,39,40,41,42,43,44,45,46,47,48,49,50,51,52,53,54,55,67,69,70,71,72,73,74,75,76,77,78,79,80,81,82,86,87,88,89,90,91,92,93,94], established-coefficients [10,11], modelled coefficients [38], mathematical coefficients [9], a logistic profile model [43] and inverted salinity profiles [56].

Johnston et al. [19] reported that EM38 readings are not highly accurate but that categories of soil salinity for large areas can be readily established. Coefficients of determination between 0.88 and 0.9 at depth levels of 30–60, 60–90 and 0–90 cm in soils in which salinity was the dominant factor influencing the EM38 readings were described by Amezketa [59]. A more complex example of these regressions is the dual pathway parallel conductivity (DPPC) model developed by Rhoades et al. [32]. This model indicates the major contribution to EC_a_ readings from conductivity in the water-filled pores that contain the majority of the solved salts with a relatively small contribution from the exchangeable cations. When comparing different EC_a_-EC_e_ prediction models, the relationships often show low accuracy [5,19,78]. These results suggest that it is essential to establish calibration relationships between EC_a_ and EC_e_ that depend on the soil type and water status for the specific site conditions for a particular survey [19,20]. The variability of EC_a_ to EC_e_ conversion is greater in coarse-textured soils than in medium- or fine-textured soils [24].

The effect of soil salinity and soil water content on the EC_a_ has been described e.g., by Hanson and Kaita [91], Bennett et al. [65], Gill and Yee [16], Turnham [81] and Wittler et al. [54]. The results indicated substantial changes in the EC_a_ readings as soil-water content changed. A linear relationship existed between soil-water content and EC_a_ for each level of soil salinity across the range of measured soil water contents [91]. Norman [30] stated that, for clay soils (i.e., >40% in the top 30 cm), the water content of the soil profile should be greater than 20% to allow soil salinity values to be accurately derived from the observed EC_a_ data. In Iranian investigations, Rahimian and Hasheminejhad [35] found that more reliable regression equations between EC_ah_ (horizontal mode) and EC_av_ (vertical mode) and soil salinity could be derived at 35% water content in comparison to 25% water content. Arndt et al. [60] cited similar values from the USDA-Soil Conservation Service. For field surveys where EC_a_ was closely related to salinity, Corwin and Lesch [97] used relationships between the v- and h-mode to derive new variables. The geometric mean (sqrt(EC_ah_*EC_av_)) provides a measure of the cumulative EC_a_ through the root zone and the ratio mean (EC_ah_/EC_av_ ) characterizes the degree of leaching. A ratio greater than 1 indicates that the net flow of water and salts is upward, and a ratio less than 1 indicates a downward net flow.

Broadfoot et al. [66] and Mankin and Karthikeyan [80] described similar classifications:

- Leached soils, where salinity increases with depth, defined by EC_ah_/EC_av_ ≤ 1.0
- Uniform, where salinity does not change significantly with profile depth and where 1.0 < EC_ah_/EC_av_ ≤ 1.05, and
- Inverted salinity profiles, where salinity decreases with depth and where EC_ah_/EC_av_ > 1.05.

A similar representation was chosen by Spies and Woodgate [93]. Subsoil (EM31) salinity maps and root zone (EM38) maps were combined to provide an assessment of salinity hazard. The EM38 instrument had a depth range of less than 1.5 m, while the EM31 probes had a depth range of 4 to 6 m. Triantafilis et al. [42] developed a leaching fraction model in combination with EC_a_ based on the amount of deep drainage and the average root zone EC_e_. However, the present investigations are not limited to the creation of real-time inventories but are also of value in forecasting temporal changes in the salinity status. Lesch et al. [100] used pre- and post-EC_a_ surveys to quantify the degree of salt removal from a field. However, the spatial variability impeded the derivations, particularly for subareas with high salinity levels. Salama et al. [101] related apparent conductivity to recharge/discharge mechanisms within watersheds. They associated low values of EC_a_ with low concentrations of total soluble salts and recharge areas. Discharge areas were associated with high values of EC_a_, indicating greater concentrations of soluble salts near the surface and inverted salt profiles. The latter were associated with rising groundwater tables, increased groundwater flow with mobilization of soluble salts, and greater discharge at or near the surface. All of these factors are related to saline seep development [102].

In an advanced application, EM38-EC_a_ was used to help to assess the salt tolerance of trees, forages and turf grasses [14,16,23,24,25,26,65]. The authors also studied the usefulness of EC_a_ to predict the survival and growth of eucalyptus and pastures in saline soils. According to McKenzie et al. [24,25] and McKenzie [26], close correlations between salinity measured as EC_a_ to the yield of wheat and salinity measured by the saturated paste extract by McKenzie [26] were equal. In contrast, relationships of EC_a_ with observations on the establishment and growth of perennial pasture species were weak [16]. Kaffka et al. [20] reported that, in locations where crop growth were influenced by salinity, EC_a_ was useful for estimating optimum N-fertilizer application and for identifying areas of the field with unprofitable yields. Horney et al. [92] developed a four-step method for site-specific salinity management in commercial fields. The steps included (1) generation of an EC_a_ map; (2) directed soil sampling for EC_e_; (3) determination of the estimated amendment requirement as a function of location in the field; and (4) integration of the individual amendment requirements into a practical spatial pattern for amendment application. As early as 1997, McKenzie et al. noticed that EM38 is a cost-effective tool for assessing field salinity and for use in experiments on the salt tolerance of crops.

Vaughan et al. [48] combined EC_e_ and water content of soil samples with field wide EC_ah_ measurements. The prediction of soil salinity at unsampled points was carried out by co-kriging of logEC_e_ with EC_ah_. In a comparison to the work of Triantafilis et al. [44] co-kriging and regression kriging of the EC_a_ readings also showed minimum errors compared to ordinary and three-dimensional kriging.

All of the cited procedures are practical only if salinity is the main factor influencing EC_a_ and if EC_e_ shows a close relationship to EC_a_ [65]. Otherwise, a multiple regression model with further independent influencing factors is required. Consequently, calibration equations and modelled results cannot be used on other sites very often.

## 4. Detecting Soil-Related Properties in Non-Saline Soils by EM-38

### 4.1. Influence of Soil Water Content Conditions

In soils where salinity is not a significant factor, EC_a_ values primarily represent as a function of soil water content and the amount of electrical charge. Many researchers recommend measurements with the EM38 at a soil water content close to or at field capacity [49,103,104].

This praxis has its basis in the theory of Rhoades et al. [32] and Corwin and Lesch [97]. In sufficiently wet soils, soil water is the major conductive pathway. Here EC_a_ is determined by the volumetric content of soil water. However, to an increasing extent of researchers noticed that the spatial patterns of EC_a_, measured under different soil water conditions, are relatively stable with time; only the level indicates a change [105]. However, the relationship between EC_a_ and soil physical and chemical properties varied considerably depending on the actual water conditions. This weak temporal stability of relationships between EC_a_ and other soil properties indicated that soil water conditions have a significant influence on EC_a_. When there is not enough water in the continuous pores, the surfaces of soil particles and the small discontinuous pores of the soil are the main pathways (e.g., when soil water content is <60 to 70% of field capacity). Under these conditions, the influence of the soil particle volume, the volume and conductivity of water in the small pores, as well as the surface- conductivity of soil particles, increases [32].

Bang [106] showed that several variables (i.e., bulk density, percentage of sand, silt, and clay, plant-available water content, cone index, and saturated hydraulic conductivity) and chemical parameters (i.e., extractable P and K, pH, cation exchange capacity, organic matter, and micronutrients) presented different strengths of the correlations with EC_a_. Few direct strong correlations were found between EC_a_ and the soil physical properties studied (*R*^2^ < 0.50), yet overall, the correlation improved when EC_a_ was measured under relatively dry conditions. Furthermore, according to Bang, the utility of EC_a_ as a variable in cluster analysis to indicate management or soil sampling zones was influenced by variations in EC_a_ measured under different soil water conditions. Bang suggested “that the spatial and temporal EC_a_ variability measured under different soil water conditions could be a critical factor when evaluating the ability of EC_a_ to predict soil chemical and physical characteristics important to soil and crop productivity and management”. Therefore, Bang [106] recommended that an EC_a_ survey be conducted under relatively dry conditions in similar coastal plain soils.

Lück et al. [107] carried out measurements on loamy fields, partly with coarse textured sediments. The authors found the most pronounced EC_a_ distributions during summer (relatively dry conditions). This may has been caused by the larger water content fluctuations in the sandy soils due to their lower water-holding capacity. In contrast to these soils, the loamy parts of the fields had a higher water content as a consequence of higher water-holding capacity as well as better water delivery via capillary rise. Conversely, at sites with dominant Pleistocene loess soils, readings taken during periods when soil water content was at field capacity produced more pronounced maps [108]. Under drier conditions, the EC_a_ readings indicated lower, more similar values. Some researchers recommend a different procedure. Mertens et al. [109] suggested the creation of an averaged map from repeated recordings made at different dates. This procedure is scientifically more appropriate than a water correction. Zhu et al. [110] indicated that the best mapping of major soil distribution across a landscape studied in Pennsylvania required optimal timing, meaning the occurrence of a wet period. No single survey or relative differences in EC_a_ obtained by repeated measurements was sufficient to obtain the best possible soil map for the study area. A combination of repeated surveys, depth to bedrock, and terrain attributes provided the best mapping of soils in this agricultural landscape and doubled the accuracy of the map. EC_a_ measurements collected during the wetter periods (i.e., >10-mm antecedent precipitation during the previous 7 days) showed greater spatial variability (i.e., greater sills and shorter spatial correlation lengths), indicating the influence of soil water distribution on soil EC_a_ [111].

### 4.2. Soil Texture

Frequently, in non-saline soils, EC_a_ is used to indicate soil texture, particularly clay content. Simulations of silt and sand are rare and seem more likely a by-product. However, the quality of the single relationships are often rather confounding (Table 2). As noted by Corwin and Lesch [112] the target variables correlate inconsistently with EC_a_ mainly as a consequence of: (1) the complex interaction of soil properties; (2) a temporal component of variability that is only weakly indicated by an expected constant variable such as EC_a_ and (3) variable climatic factors.

McBratney et al. [113] and McBratney and Minasny [114] demonstrated that differences in the mineral composition influence the magnitude of the EC_a_ values and therefore the strength of the relationship to the clay content. Kaolin-dominant soil minerals will have smaller conductivities, and soil that mainly contains illite or has a mixed mineralogy will have larger EC_a_ values, but these values are smaller than those for smectitic materials. Furthermore, the authors noticed that at low conductivities (<50 mS m^−1^), it is quite difficult to separate clay. Wayne et al. [115] derived texture fineness classes from EC_a_ readings. A conductivity greater than 30 mS m^−1^ indicated clay, and a conductivity less than 5 mS m^−1^ indicated sand. Furthermore, EC_a_ values between 0 and 10 were classified as sandy loam, and values between 10–20 mS m^−1^ indicated clay loam. These fineness classes represented a basis for the derivation of the plant-available water content. Domsch and Giebel [116] described another approach to delineate clay content. Working with predominantly sandy soils, the authors indicated that, at field capacity, EC_a_ reflected this property well. However, for soils with water-influenced horizons (gleyic soils), such relationships are very weak and should not be introduced in calculations for mineral soils. A factor scoring that used clay and silt content showed a closer relationship with EC_a_. Furthermore, the authors related EC_a_ to soil textural classes: an EC_a_ of 0–10 mS m^−1^ indicated sand or loamy sand, an EC_a_ of 10–20 mS m^−1^ indicated sand or loamy sand over loam, and an EC_a_ of 20–30 mS m^−1^ indicated sandy loam or loam. Vitharana et al. [104] used the geometric mean ((EC_av_·EC_ah_)^0.5^) to delineate the clay content of the top- and subsoils. Doolittle et al. [117] used EC_a_ to locate small inclusions of sandy soils within a predominately fine-textured alluvial landscape. Bobert et al. [103] improved the relationships between EC_a_ and clay, silt and clay + silt by extracting the drift caused by soil water content calculated from a wetness index map. A multi-site/multi-season approach to calibrate EC_a_ models for predicting clay content across large landscapes was developed by Harvey and Morgan [118]. The fact that the relationships between clay and EC_a_ were similar in all 12 fields, indicated that a single linear regression model could be used to describe the spatial variability of the clay content across all of the fields. This “single calibration approach” used data from a designated calibration area to estimate EC_a_ model parameters that were then combined with data from subsequent fields to predict the soil variability in the observed fields. The single calibration approach is likely applicable to other areas, providing requirements for its use are met. Those requirements include the following: (1) the distribution of the soil property or properties of interest in calibration area should be representative of the study area; (2) the soil property or properties that influence EC_a_ should be the same across the study area; and (3) management practices (e.g., crop rotation and irrigation) should be similar across the study area.

To an increasing extent, methods other than linear regression have been used. Response surface sampling design, fuzzy k-means classification, hierarchical spatial regression modelling and EC_a_ (EM38 and EM34) surveys were applied by Triantafilis and Lesch [119] to produce a map of spatial clay content. Triantafilis et al. [44] combined EC_a_ values (EM38 and EM31) and clay content with different geostatistical methods (co-kriging, regression-kriging and ordinary-kriging). The results suggested that the linear relationship of clay content against EC_a_ (EM38) data used in combination with kriging of regression residuals was the most accurate. Vitharana et al. [104] showed that standardized ordinary kriging of subsoil clay content as the primary variable and the geometric mean ((EC_av_*EC_ah_)^0.5^) as the secondary variable gave better results when compared to ordinary kriging and traditional ordinary kriging.

### 4.3. Soil Water Content, Water Balance

The derivation of the water storage capacity, particularly the field capacity, and the plant-available water content based on electrical conductivity measurements has gained increasing importance. Table 3 provides an overview of current investigation areas and target variables.

Water content, like salinity, is a horizontally and vertically effective dynamic property. In areas where water content is the dominant factor that influences EC_a_ and where water content decreases with depth, EC_ah_ > EC_av_ and vice versa [167]. Wayne et al. [115] developed a hierarchical procedure for calculating available water content. EC_a_ was used to target the location for neutron probe samples. The construction of a water content–texture relationship allowed the determination of the available water content and the soil water deficit. Kachanoski et al. [141] found that in soils with a low electrolyte content and a wide range of texture, EC_a_ explained more than 90% of the water content. Additionally, Kachanoski et al. [142] correlated EC_a_ readings with water storage and found that 50–60% of the variations in EC_a_ were explained by water content. Similar levels for coefficients of determination were described by Sheets and Hendrickx [150] and Khakural et al. [143]. Morgan et al. [147] noted that EC_a_ is only applicable in areas with a greater range of water content. The same observation was made by Hedley et al. [135],who calculated an *R*^2^ of 42%. Substantial changes in the relationships between EC_a_ readings and soil water content were shown by Hanson and Kaita [91]. The higher the soil salinity was, the more sensitive the EC_a_ readings were to changes in soil water content. A linear relationship existed between soil water content and EC_a_ for each level of soil salinity over the range of measured soil water contents. In a Mollic catena, Brevik et al. [139] found significant relationships between EC_a_ and soil water content that explained 50% to over 70% of the variability. The greatest difference between EC_a_ values in any soils was observed when the soils were moist. Regression line slopes tended to be lower in higher landscape positions indicating greater EC_a_ changes with a given change in soil water content. A relationship between increasing water content and EC_a_ readings from a summit-to-foot slope area of calcareous till parent material with a coefficient of determination of 0.86 was described by Clay et al. Wilson et al. [161,162] derived areas with different water movements from EM31 and EM38 readings. Drying/draining patterns were characterised by a downward shift in EC_a_ with time. Follow-up EC_a_ surveys across high-to-low patterns showed a positive correlation between EC_a_ and water content. Regions with increased horizontal flow showed high conductivities after rainfall. Areas that had preferential vertical flow showed lower EM38 readings after periods of rainfall. For a prototype engineered barrier soil profile designed for waste containment, Reedy and Scanlon [148] and Reedy [149] predicted the average volumetric water content at any location at any time with a linear regression model (*R*^2^ = 0.80) and spatially averaged volumetric water contents over the entire area (*R*^2^ = 0.99).

Bang [106] described weak and negative relationships between soil water content and EC_a_ values in North Carolina’s Coastal Plains. Little variation in subsoil water content across the study site for each survey date combined with a relatively narrow range of variability in soil texture was the main reason for this result. Furthermore, the variability in other factors (e.g., soil compaction and texture) might have masked the contribution of the water content to EC_a_ variation., The author concluded that the spatial variability of soil water content at a 0- to 75-cm depth could not be directly determined by a field-scale EC_a_ survey at this site, due to the weak relationships between soil water content and EC_a_. Relationships between plant-available water content and EC_a_ (*R*^2^ = 0.78) were derived by Wong and Asseng [152] to transform a water storage capacity map of the field into yield maps for three major season types (dry, medium and wet) and nitrogen fertilizer management scenarios. Hall et al. [159] reported that EC_a_ methods (i.e., EM38 and the use of a borehole conductivity meter) could accurately characterize water and solute distributions in the vadose zone. Saey et al. [168] developed an index to register the area-wide soil heterogeneity. After calculating the relationship between clay content and EC_a_, this equation was converted so that EC_a_ was the target variable. In the next step, the authors calculated a quotient of the measured EC_a_ and the EC_a_ reading derived from the clay content. This result was called EC_ref_ and was used as measure for soil heterogeneity.

Variables other than water content are targets of EC_a_ measurements to an increasing extent; for example, hydraulic conductivity, water table depth, drainage classes and groundwater recharge. In developing a relationship between EC_a_ and estimated deep drainage (mm/year) Triantafilis et al. [42,45] developed four-parameter broken-stick models fitted to EC_av_ beyond 120 cm. Vervoort and Annen [163] showed that the overall patterns of the hydraulic conductivity of palaeochannel in alluvial plains could be inferred from the combination of EM inversion using EM38 and EM34 measurements. However, the absolute magnitude of hydraulic conductivity could not be easily predicted.

Sherlock and McDonnell [169] used simple linear regression analyses to compare terrain electrical conductivity measurements from EM31 and EM38 to a distributed grid of water table depth and soil- water content measurements in a highly instrumented 50 by 50 m hill slope in Putnam County, New York. Regression analysis indicated that EC measurements from the EM31 meter (v-mode) explained over 80% of the variation in the water table depth across the test hill slope. Despite problems with sensitivity and zeroing the EM38 could explain over 70% of the gravimetrically determined soil water variance.

The depth of the water table was also detected by Schuman and Zaman [160]. Knowledge of the water table depth was necessary to select a suitable field for new citrus plantings and for drainage systems. With EC_a_ in the vertical mode, the authors could estimate these values with a RMSE of approximately 4–15 cm. EC_a_, the topographical wetness index and the rainfall time series gave good predictions of water content and water table depth using the models derived according to Hedley et al. [140]. Further investigations determined soil drainage classes [144], groundwater recharge [170], water drainage [46] and irrigation [164].

### 4.4. Detection of Soil Horizons and Vertical Discontinuities

To an increasing extent, investigations were carried out to calculate EC_a_ depth profiles in combination with the detection of vertical discontinuities (Table 4). Refining and improving of soil maps is necessary for soil protection and the description of soil functions.

EC_a_ profiling by depth requires more intensive measurements. Usually, this investigation is carried out with measurements made at different heights above the soil surface or repeated measurements at different coil spacing using regressions between EC_a_ and depth for the further calculation [5,9,185,212]. As the instrument is raised above the ground, the relative influence of deeper layers on the measurements decreases. Visual comparison of EC_a_ values and instrument height and inverse modelling (inversion, optimization) are often used. However in numerous cases, the alternating influencing factors impede the retrieval of adequate results; for example, both texture and salinity can cause strong vertical fluctuations. Sudduth et al. [196], Sudduth and Kitchen [155,175,176,177,178,179,181,184,185,186,187,188,195,196,197,198,201,202,203,204,205,206,207,208,209], Kitchen et al. [213] and Noellsch [214] used EC_a_ to determine the depth to the claypan (the sublayer with 50 to 60% clay, varying in depth from 0.1 to 1 m) in nonsaline soils (Missouri). A high correlation between increasing EC_a_ and decreasing depth to the claypan was observed by Doolittle et al. [184]. The depth of boulder clay was estimated by Brus et al. [193], and Bork et al. [191] estimated the loess thickness above basalt. Mapping of sand deposition after floods was carried out by Kitchen et al. [187]. In the investigations of Boettinger et al. [190] soil depth to the petrocalcic horizon was positively and significantly correlated with EC_a_. Doolittle and Collins [183] reported that bedrock depths on a Pennsylvania site, based on depth classes, could be estimated with EC_a_ data.

Knotters et al. [188] introduced EC_a_ as an auxiliary variable in co-kriging and kriging with regression to predict the depth of Holocene deposits. Vitharana et al. [189] improved the content of a soil map with the calculation of the depth of a Tertiary stratum.

### 4.5. Relationships to N-turnover, Cation Exchange Capacity, Organic Matter and Additional Soil Parameters

In addition to the previously listed soil properties, further parameters have been combined with EC_a_ readings, including cation exchange capacity, organic matter, bulk density, nutrients (e.g., NO_3_^−^, Olsen-P) and elements such as Ca, Mg, K, Na (exchangeable or in saturation extract), B, Mo, H and other anions. For close relationships, field-wide ECa measurements allow mapping of soil properties (Table 4). The dominant target variable was the cation exchange capacity [3,132,135].

The leaching rates calculated from a field study were related to changes in EC_a_ readings [209]. This enabled the derivation of a spatially averaged leaching rate. The spatial distribution of N seems to be an increasingly attractive parameter to be estimated via soil conductivity. Eigenberg and Nienaber [215,216] and Eigenberg et al. [217,218] related EC_a_ maps made at different times to temporal values of available N and other specific mobile ions that were associated with animal waste and cover crops, and concluded that EC_a_ can be used as an indicator of the content and loss of water-soluble N. Eigenberg and Nienaber [215,219] isolated and detected areas of nutrient build-up in a cornfield receiving waste. Different manure and compost rates had been applied for replacement of commercial fertilizer. EC_a_ measurements differentiated commercial N-fertilized plots from those that had manure applied at the recommended P rate, compost applied at the P rate, and compost applied at the N rate. In another publication, the same authors [220] discriminated areas with synthetic fertilizer from areas with feedlot manure and compost application. Differences between EC_a_ maps before and after the applications were partly explained by N decompositions. Furthermore, Eigenberg et al. [221] reported that EC_a_ (EM38 and Dualem-2) soil conductivity appeared to be a reliable indicator of soluble N gains and losses in a soil under study in Nebraska, a measure of available N sufficiency for corn mainly in the early growing season, and an indicator of NO_3_^–^N surplus after harvest when soluble N was vulnerable to loss as a consequence of leaching and/or runoff.

Johnson et al. [204] stated that in soils where EC_a_ is dominated by NO_3_^−^-N, EC_a_ was applicable for tracking spatial and temporal variations in crop-available N (manure, compost, commercial fertilizer, and cover crop treatments). Furthermore, the calculation of fertilizer rates for site-specific management was possible. Stevens et al. [222] studied EC_a_ as an indirect measure for NH_4_^+^ and K^+^ in animal slurries. The predictive capability of soil conductivity to estimate soil nitrate was demonstrated by Doran and Parkin [223]. Korsaeth [125] found an explanation of a variance of 27–69% (average 47%) of topsoil inorganic N concentration by means of EC_a_. In general, the author stated that determination of absolute levels of this parameter was difficult with EC_a_, but it appeared to be quite a robust method for detection of both spatial and temporal relative differences. Some authors described relationships between ECa and soil conditions that influenced soil mineral N [224,225]. Fritz et al. [224] suggested the application of ECa to predict NO_3_**^−^** concentrations in the soil. A comparison of the EM38 and the Veris 3100 sensor cart showed a correlation with soil NO_3_^−^, but the authors indicated that further studies were necessary to confirm their results.

The studies of Jaynes et al. [226] and Kitchen et al. [213] assumed a possible relationship between soil ECa and N mineralization and denitrification rates. Soil conditions, especially the texture, influenced the rate of denitrification and N mineralization [227]. The relationships between soil texture and N mineralization and denitrification should aid in developing an in-season variable-rate N fertilizer recommendation [224]. Soil organic matter, ECa, and soil texture are properties that might aid in predicting mineralization and denitrification in soil. Dunn and Beecher [228] detected large differences in surface soil acidity and a strong relationship (*R*^2^ = 0.49 to 0.91) compared to EC_a_ readings in individual rice fields in NSW, Australia. The proposed EC_a_ levels for the delineation of zones were *<*80, 80–140 and *>*140 mS m^−1^ for the EM31 vertical mode, and *<*80, 80–110 and *>*110 mS m^−1^ for the EM38 vertical mode. Many rice growers in southern NSW currently have EM maps of their fields. Using these maps soil sampling for soil acidity would be a more cost-effective method than grid sampling.

Triantafilis and Momteiro Santos [200] indicated the cation exchange capacity (CEC) as one of the most important soil properties because it is an index of the shrink–swell potential and is thus a measure of soil structural resilience to tillage. The authors used the readings from EM38 and EM31, and additionally remotely sensed spectral reflections (red, green and blue spectral brightness), and two trend surface (Easting and Northing) variables as ancillary data or independent variables, and a stepwise MLR model was used to predict the CEC. The x and y variables accounted for any distinct drift in the residual error pattern. The correlation coefficient (*R*^2^ = 0.76) for the regression model was much larger than that achieved with any of the individual ancillary data variables. The adjusted *R*^2^ was 0.69, and the estimated RMSE was 1.86 cmol kg^−1^.

In other studies, the results were more confusing. Heininger et al. [229] and Nadler [230] indicated that salinity, soil texture, or soil water content were masking the response of EC_a_ to other physical, chemical and nutrient levels in soil. Cations, such as Ca, Mg, or K, commonly associated with binding sites on soil particles, could influence EC_a_ with variations in EC_S_ (i.e., conductivity of the solid soil). However, the common assumption is that in most field solutions, changing levels of soil cations have a minor influence on EC_S_ [229,231]. Heininger and Crosier [232] demonstrated that under saturated conditions changes in nutrient levels (e.g., soluble N and S), changes in EC_WC_ could influence EC_a_. In a study by Heiniger et al. [229], EC_a_ was evaluated as a means to estimate plant nutrient concentrations (i.e., P, K, Ca, Mg, Mn, pH, CEC, and humic content). This study indicated that it was unlikely that ECa could be used to directly estimate the soil nutrient content in a field. However, the authors suggested that additional research on the relationships of EC_a_ with soil water content and soil texture was necessary to determine whether EC_a_ could be used to establish nutrient management zones. The authors concluded that “EC_a_ can be valuable tool when used in conjunction with multivariate statistical procedures in identifying soil properties and their relationship to nutrient availability”.

According to Martinez et al. [203], EC_a_ can provide inexpensive and useful information to capture soil spatial variability and characterization of organic carbon. EC_a_ data were used to elucidate differences in soil properties as a consequence of topography and management, explaining >25% of the spatial variation. With normalized EC_a_ (ΔEC_a_) the authors successfully applied fuzzy k-means to delimit homogeneous soil units related to soil management and the spatial distribution of organic carbon. Grigera et al. [131] related soil microbial biomass to organic matter fractions in a field using EC_a_. Soil properties (0–90 cm) that showed higher correlations with EC_av_ (C_t_ (*R* = 0.87), clay (*R* = 0.83), total dissolved solids (*R* = 0.68), and depth of topsoil (*R* = 0.70)) influenced soil water availability in this field. Soil microbial groups were correlated with different soil C fractions in the uper 15 cm and were similar across EC_a_ zones. Motavalli et al. [233] assessed variation in soil Bray 1 P levels in litter amended landscapes at 0–5 and 5–15 cm depths. EC_a_ was also applied as subsidiary variable in a co-kriging method for improving the map accuracy interpolation of P, K, pH, organic matter and water content [210]. Jung et al. [207] described a similar effect for the application of EC_a_. Cross-semivariance analysis with EC_a_ as a secondary variable were better than by a simple semivariance analysis.

Bekele et al. [234] reported that EC_a_ was strongly related to ammonium extractable K, organic matter (OM), pH and Bray-2 phosphorus with factor analysis but not to ammonium extractable Ca and the sum of bases in fields in LA, USA. Furthermore Lukas et al. [127] examined soil chemical characteristics (i.e., P, K, Mg content and pH value) and humus content and showed relatively balanced, moderately strong correlations with EC_a_.

Additionally, the use of EC_a_ for the detection of soil compaction has become increasingly important [192,208]. Krajco [208] discovered that the EC_a_ readings measured in the horizontal mode distinguished the areas with no compaction above 0.3 m and areas with soil compacted in the entire soil profile with less precision. The EM38 operated in the vertical mode was not sensitive enough to measure any differences in soil bulk density.

### 4.6. Derivation of Soil Sampling Designs

EC_a_ measurements are frequently applied to devise soil sampling schemes to reduce soil sampling points (Table 5) [88,114,115,235,236].

In addition to finding representative locations, the goal is to significantly reduce the number of samples required to effectively calculate the target variable. Frequent selection of sampling points by means of EC_a_ surveys is performed empirically. In principle, design-based (probability-based) and model-based (prediction-based) sampling schemes are applicable.

Triantafilis et al. [42,45] used the ratio (EC_av_(EM38)/EC_av_(EM31)) to determine soil sampling points on salt affected areas. Lower ratios appeared when EM38 was sensing the relatively sandy and less conductive topsoil. The results of Shaner et al. [243] support the utilization of EC_a_-directed zone sampling as an alternative to grid sampling if the transition zones of soil texture and soil organic matter are avoided. Approximately 80% of the samples in grid sites 10 m from the zone boundaries were classified correctly compared to the samples <10 m from the boundary, in which only 50–54% were classified correctly. Corwin et al. [237] described a procedure that was the basis for the development of the ESAP software package [240,241]. In this model-based sampling approach, a minimum set of calibration samples was selected based on the measured ranges and spatial locations of the EC_a_ readings. This sampling approach originated from the response surface sampling design (RSSD) methodology of Box and Draper [244]. The ESAP software was specifically designed for use with ground-based EM signal readings. The ESAP software package tried to identify the optimal locations for soil sampling (6–20 sites depending on the level of variability of EC_a_) by minimizing the mean square deviation. Zimmermann et al. [235] developed a hierarchical system with (1) EC_a_ measurements; (2) kriging; (3) cluster analysis; (4) principal component analysis and (5) formation of a pseudo-response surface design to select subsets of appropriate sites for soil sampling. The number of samples could be minimized while still retaining the prediction accuracy inherent in statistical sampling techniques. Horney et al. [92] suggested a methodology for salt affected soils with the following steps: (1) building an EC_a_ map; (2) directed sampling for salinity; (3) as a function in the field determination of the estimated improvement requirement and (4) integration into a practical spatial pattern. Tarr et al. [245] used stratification of EC_a_ and terrain attributes to derive a heterogeneous pasture in relatively homogenous sampling zones with fuzzy k-means clustering. The five zones had significant differences in the target variables (i.e., P, K, pH, organic matter and water content). However, the reduction of sampling points from 116 to 30 to 15 points resulted in a loss of accuracy, but this loss may not have an economic or management consequence to the producer. Yao et al. [242] described a completely new method based on Minasny and McBratney [246]. The authors developed the application of the VQT (variance quad-tree) method on sampling design with the digital elevation model and its derivatives and Landsat TM images. EC_a_ was selected as an additional variable, and the spatial distribution map of EC_a_ was used as design detecting salinity. The results show that the spatial distribution of soil salinity detected with the VQT scheme was similar to that produced with grid sampling, while the sample quantity was reduced to approximately one-half. The spatial precision of the VQT scheme was considerably higher than that of the traditional grid method with respect to the same sample number. Fewer samples were required for the VQT scheme to obtain the same precision level. The authors suggested that VQT and EC_a_ provide an efficient tool for lowering sampling costs and improving sampling efficiency in the coastal saline region.

### 4.7. Derivation of Soil Type Boundaries

Delineating soil classifications has quite different levels of complexity and accuracy. ECa is applied to support the derivation of soil types (Table 4). Very often, the first question concerns the interpolation of the EC_a_ procedure. Niedźwiecki et al. [247] gave an overview of EC_a_ field-wide variability with variograms. The authors recommended an individual interpolation because of differing variability between fields. Selection of parameters for semivariograms has a strong influence on the ability to identify significant spatial autocorrelation of data. Lag parameter size and directional analysis of variance are particular concerns.

The next question concerns the interpolation of EC_a_ across field boundaries. As a consequence of land use, time of measurement, wetness, and fertilization differences between single fields, considerable differences in the EC_a_ levels frequently exist. Weller et al. [121] presented a method for unifying EC_a_ across boundaries with a “nearest-neighbours EC_a_ correction”. EC_a_ measurements near field boundaries were correlated with EC_a_ values of the neighbouring field, resulting in the same level of EC_a_ in both fields. This procedure also enhanced the coefficients of determination.

**Figure 3:**
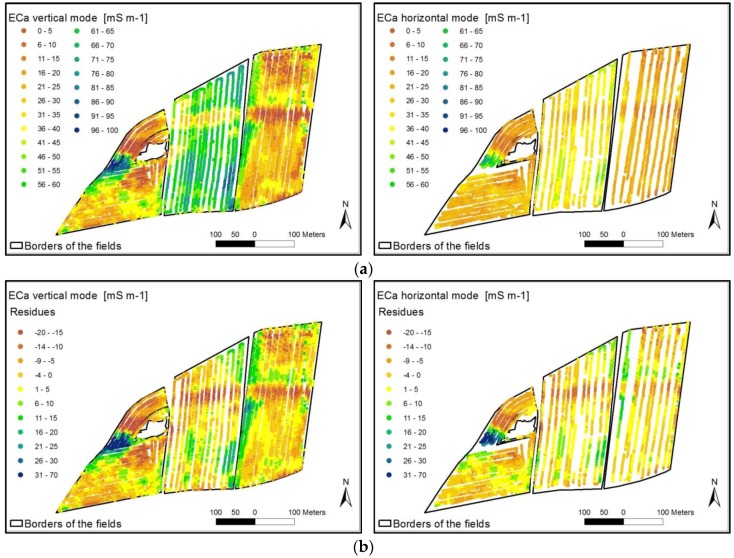

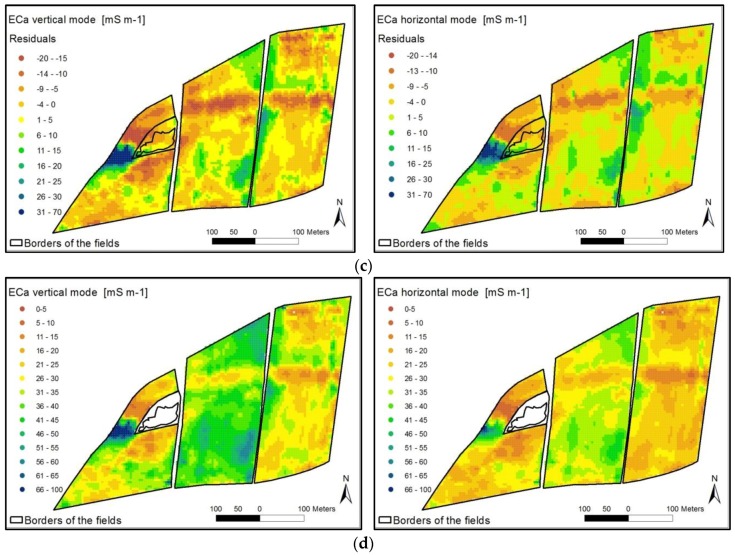
Procedure of interpolation of EC_a_ across field boundaries. (**a**) Lanes of EC_a_ –measurements with EM38 on arable farmland (16.9 ha); (**b**) Lanes of EC_a_ –measurements (field-by-field means (m_field_) were subtracted from individual observations); (**c**) Interpolation 5 m × 5 m grid of ECa (residuals); (**d**) Interpolation 5 m × 5 m grid of ECa (residuals+local means).

Another procedure was described by Heil and Schmidhalter [108] (Figure 3). To reduce the levels and to obtain reliable EC_a_ values across field boundaries, the following steps were used: (1) The field-by-field means (m_field_) were subtracted from individual observations (Figure 3b); (2) The resulting new EC_a_ (z_residual_) values were then used as input to estimate the residual variogram. The EC_a_ data were interpolated, and continuous maps of EC_a_ residuals were obtained (Figure 3c); (3) Finally, the field-by-field means (m_field_) were added back to the estimated point-kriged surfaces (z_krig_) for each particular field (Figure 3d). With this procedure it is possible to interpolate point wise or row wise measurements with a single interpolation calculation.

Nehmdahl and Greve [128] compared soil profile descriptions and interpolated EC_a_ measurements to derive areas with more or less similar soil types. Stroh et al. [181] distinguished boundaries of soil map units in a relative manner. In different instances, gradients or contrasting inclusions within map units were also identified. In this investigation, correlations between EC_a_ readings and soil properties such as CEC, pH, particle size distribution and extractable bases were low (i.e., explained <6% of the variance) or non-significant. James et al. [178] used confusion matrix analysis to determine whether EC_a_ and a clustered k-means algorithm accurately delineated soil textural boundaries in a field containing clay loam and sandy loam soils. The agreement between the EC_a_ data and the two soil classes was 62%. Hedley et al. [135] derived two soil units (clayey soils and silty loamy soils) with a discriminant analysis of an EC_a_ survey. A more detailed prediction was not possible.

Often, the use of EC_a_ is restricted to its application as covariate or the readings are used in a relative sense, not as absolute terms. In some studies, combination with further predictors such as terrain attributes or yield deliver an acceptable result [179]. Rampant and Abuzar [179] predicted soil types from the various combinations of geophysical (EM38, EM31, airborne gamma radiometrics) and terrain attributes with a decision tree classifier. Individually, the geophysical data were relatively weak predictors of soil information. Using all of the geophysical and terrain data, the soil types were predicted very well, with less than 2% of the area misclassified. Clay et al. [248] empirically derived soil patterns from EC_a_ readings and elevation data. Generally, well-drained soils in the summit area and poorly-drained soils in the valley bottoms had low and high EC_a_ values, respectively.

An interesting comparison between EC_a_ and the soil values of the German national soil inventory (Bodenzahlen) was presented by Neudecker et al. [249]. In 11 fields in four different German regions, *R*^2^ varied between 0.1 and 0.71. Highly heterogeneous fields showed a range of *R*^2^ values from 0.03–0.71. The authors concluded that EC_a_ measurements were much better in delineating zones of different soil substrates than other, rather subjective methods such as the German national soil inventory.

## 5. Applications in Agriculture

### 5.1. Derivation of Agricultural Yield Variability and Management Zones

EC_a_ is used to reflect crop yields and to derive management zones. Different studies show that crop yields vary due to site-specific differences and temporal climatic changes (Table 6).

**Figure 4:**
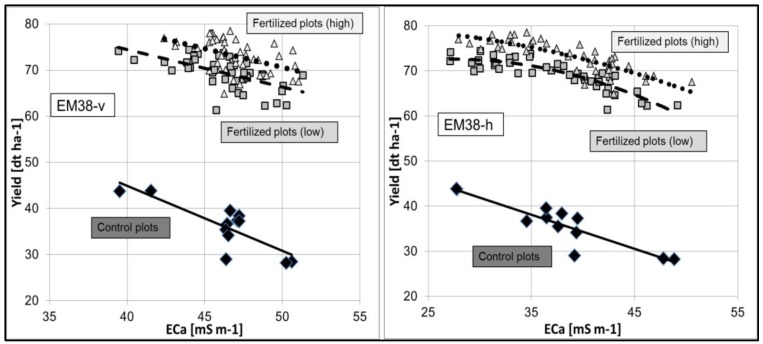
Relationships between EC_a_ and multi-annual mean of yield (wheat) of the long-term experiment Dürnast 020 in dependence of fertilization level (control plots: no fertilizer, fertilized plots (low): 100–140 kg ha^−1^ N, fertilized plots (high): 150–180 kg ha^−1^ N).

Management (productivity) zones with similar yields and used by farmers to make application decisions based upon calculations of the expected yield. The applied methods and additional predictors are different in this context. In fact, EC_a_ has no direct relationship to the growth and yield of plants, but the spatial variation of EC_a_ is partly correlated with soil properties that do affect crop productivity. Several studies have shown this connection [88,127,213,226,271]. The advantage of EC_a_ in comparison to yield measurements is its relative temporal stability, which offers a better basis for the delineation of management zones than variable yield mapping information does. With cluster analysis, Fleming et al. [258] confirmed that management zones represented different suites of soil. In one field, soil organic matter, clay, nitrate, potassium, zinc, EC_a_ and corn yield data corresponded to the levels indicated by the management zones. In a different field, only the medium productivity zone had the highest values for these parameters. Cockx et al. [253,254] used the spatial distribution of NO_3_^−^ in addition to EC_a_ to create nitrogen management zones. The interpolated EC_a_ measurements were the input for a fuzzy k means classification. This procedure placed each single point in a membership in each class [46]. The method minimized the multivariate within-class variance, and consequently, individuals in the same class had similar attributes [283]. Using a principle compound analysis, (PCA) Vitharana et al. [189,281] detected the importance of pH, EC_a-v_ and organic matter as independent key variables to characterize overall soil variation. The authors identified and delineated four classes (with a fuzzy k-means algorithm) with these variables. Clear differences in soil properties and landscape positions were found between these classes, and the three-year average standardized yields (grain and straw) were also different across the classes. Schepers et al. [277] aggregated brightness images, elevation, EC_a_ and yield into management zones using principal component analysis in combination with unsupervised classification. Domsch et al. [257] correlated EC_a_ and yield within the boundary lines method. In this context, Corwin et al. [284] combined EC_a_ with leaching of pollutants and Johnson et al. [204] combined EC_a_ with soil quality parameters (measured as bulk density, water content, clay content, organic matter, N, extract-able P, pH, microbial biomass C and N, potentially mineralizable N). In an investigation on claypan soil, Sudduth et al. [196] described a negative relationship between EC_a_ and grain yield in a dry year. The correlations with corn and soybean in a wet year in topographically highly variable landscape were also negative, as observed by Jaynes et al. [226,266]. However, in both studies no significant relationships were observed in years with a more normal water supply. In a newer study of claypan areas, Jung et al. [132] described negative relationships for corn and soybean in years with more than 150 mm precipitation, while in contrast, EC_a_ was positively correlated in years with less than 150 mm precipitation. In both cases, the correlation coefficients were not higher than 0.74. However, the authors concluded, “while correlation analysis itself is far from a definitive analysis, we suspect this similar pattern (between EC_a_ and yield) in correlation is not coincidental”. Kitchen et al. [213] related EC_a_ to yield applying boundary line analysis on claypan soils. A significant relationship (boundary lines with *R*^2^ > 0.25 on most areas) was apparent, but climate, crop type, and specific field information was also necessary to explain the structure of the potential yield by EC_a_ interaction. The authors divided the relationships between productivity and EC_a_ into four categories: (1) positive; (2) negative; (3) positive in some portions of the field and negative in others; and (4) no relationship. The strongest relationships were negative, reflecting the tendency of claypan soils to be water-limited for crop production in the majority of growing seasons [133]. Figure 4 and Table 7 show the relationships between EC_a_ (EM38 in both configurations) and yield of the long-term field experiment Dürnast 020 (South Germany, (4477221.13E, 5362908.78N), Heil, unpublished).

Here the application of different N-fertilizers with two fertilization levels has been tested since 1979. In the Figure 4 the multi annual means of the yields of wheat (1980, 1983, 1986, 1989, 1992, 1995, 1998, 2001, 2004, 2007, 2010, 2012) were divided in the two fertilization levels and the unfertilized control plots. Within this site, soils were mapped as deposits of Pleistocene loess, and the dominating soil types were fine-silty Dystric Eutrochrept and fine-loamy Typic Udifluvent (German Soil Survey, Bodenkundliche Kartieranleitung 2005). On this productive field (plant available water capacity 250 mm until 100 cm depth , C-content: 1.4% (0–30 cm) and 0.4% (50–75 cm)) all relationships are negative with always significant *R*^2^ and also linear or weak quadratic curves. Remarkable is that the curves have similar slopes, at least in the higher ECa range. The always lower coefficients of determination in the case of the vertical configuration could reflect, that the deeper soil is less important to the plant growth.

After a first visual inspectation the lowest values of yield correspond with higher contents of clay. The curve progressions allow further interpretations:

- The spatial distribution of the yield was at first influenced by the EC_a_ across the field. Treatment effects (fertilizing level, fertilizer form) were overlain by soil conditions with different EC_a_ values.
- The height of the yield was secondly assumedly determined by the level of fertilization.

In claypan soils, Fraisse et al. [260] also used a combination of EC_a_ and topographic features (with unsupervised classification) to develop zones and evaluated their ability to describe yield variability. By dividing a field into four or five zones based on EC_a_, slope, and elevation, 10% to 37% of corn and soybean yield was explained. In this context, Fridgen et al. [262] described software with a similar derivation of the subfield management zone. Kitchen et al. [285] used unsupervised fuzzy-k-means clustering to delineate productivity zones with EC_a_ and elevation measurements on claypan soils. Productivity zones were also derived by Jaynes et al. [267] based on a series of profiling steps in combination with cluster analysis to determine the relationship between yield clusters and easily measured terrain attributes (i.e., slope, plane curvature, aspect, depth of depression) and EC_a_. In contrast to the previous investigations, Kilborn et al. [269] found no strong relationships between elevation, slope, and soil EC_a_ with respect to biomass yield and composition. The results of Bang [106] indicate that clustering with EC_a_ and NIR surveys could be used to delineate management zones that characterize spatial variations in soil chemical properties. However, these zones were less consistent for characterizing spatial variability in yields across temporal water content variation. Furthermore, the author reported that clustering zones developed from EC_a_ values measured under relatively dry conditions were particularly effective in partitioning the spatial variability of SOM. It is clear that zones developed from clustering elevation and bare-soil NIR radiance were more effective than EC_a_ alone in capturing variability in K, CEC, and SOM. Clustering on EC_a_ with elevation and NIR provided better zones for these parameters and somewhat reduced the variability associated with measuring EC_a_ under different soil water conditions [106].

A similar praxis was used by Schepers et al. [277]; Chang et al. [252] and Fridgen et al. [262]. Cluster analysis of an EC_a_ map alone or with auxiliary data, such as terrain attributes and bare-soil images, has been widely used to delineate soil-based management zones. The relationship between EC_a_ measurements, soil properties and sugar beet yields in salt-affected soils was studied by Kaffka et al. [20]. In these soils, yield was most highly correlated with salinity. This work demonstrated the utility of relationships between EC_a_ and crop yield to answer resource input questions. Rampant and Abuzar [286] predicted yield zones from a combination of geophysical (i.e., EM38, EM31, airborne gamma radiometrics) and terrain attributes with a decision tree classifier. Individually, the geophysical data were relatively poor predictors of the yield zones. The combination of all sensors and terrain data could predict yield zones quite well, misclassifying only 5% of the area. The predictions of yield for an individual year were always worse for yield zones.

The purpose of the application of the EM38 by Guretzky et al. [263] was to examine the relationship of the relief parameter “slope”, EC_a_, and legume distribution in pastures. The authors concluded that slope and EC_a_ data were useful in selecting sites in pastures with higher legume yield and showed a potential for use in site-specific management of pastures. Dang et al. [12] used an interesting procedure for identifying management zones on a salinity-affected field. Two surveys of EC_a_ measurements were carried out; the first used a relatively wet soil profile (April–May 2009) to represent the drained upper limit of soil water, and the second used a relatively dry profile (October–November 2009) to represent the lower limit of soil water content extraction following the harvest of the winter crop. The authors developed a framework to estimate the monetary value of site-specific management options through: (1) identification of potential management classes formed from EC_a_ at lower limit of soil water content; (2) measurement of soil attributes generally associated with soil constraints in the region; (3) grain yield monitoring; and (4) simple on-farm experiments.

Islam et al. [264] estimated key properties to identify management zones on loess and sandy soils. The authors identified EC_a_, topsoil pH, and elevation as key properties, which were used to delineate management classes and to construct an excellent multiple regression model between yield and the key properties. Additionally, Islam et al. [265] described the construction of waterproofed housing for the EM38, which was built using PVC pipes for swimming in a paddy rice field. The EC_a_ data were classified into three classes with the fuzzy k-means classification method. The variation among the classes was related to differences in subsoil bulk density. The smallest EC_a_ values representing the lowest yield and also the lowest bulk density.

There was also a significant difference in rice yield among the EC_a_ classes, with Vanderlinden et al. [280] carried out a procedure for characterizing a management system. EC_a_ patterns expressed as relative differences (ϑ_ij_) were associated with topography, soil depth and soil structure, and the authors derived management zones with principal component analysis.

A very detailed insight into the relationship between EC_a_ and yield was given by Robinson et al. [275] for sites in Victoria, Australia. However, the multi-year measurements of yield and EC_a_ delivered an inconsistent picture. Significant influences of EC_a_ on yield were found for all measurements, but they evidenced alternating directions in semi-arid and rainy environments. (1) Decreasing yield was combined with increasing EC_a-v_ when texture-contrast and gradational soils with shallow topsoils occurred along with increasing clay content and physio-chemical constraints; (2) In soils without significant texture-contrast, in which physio-chemical conditions were more favourable for water in the subsoil, higher yields resulted; (3) Positive trends of EC_a_ and yield were attributed to the occurrence of higher plant-available water in the root zone in high and moderate yield zones. However, the *R*^2^ did not exceed 0.15 for all calculations.

Additionally, the EM38 has been applied in vineyards for describing soil variability to an increasing extent [5,15,16,17,18,19,20,21,22,23,24,25,26,27,28,29,30,31,32,33,34,35,36,37,38,39,40,41,42,43,44,45,46,47,48,49,50,51,52,53,54,55,56,57,62,70,71,72,73,74,75,76,77,78,79,80,81,82,86,90,91,92,93,95,96,97,98,99,100,101,102,103,104,105,106,107,108,109,110,111,112,113,114,115,116,117,118,119,120,121,122,123,124,125,126,127,128,129,130,131,132,133,134,135,136,137,138,139,140,141,142,143,144,145,146,147,148,149,150,151,152,153,154,155,156,157,158,159,160,161,162,163,164,165,166,167,168,169,170,171,172,173,174,175,176,177,178,179,180,181,182,183,184,185,186,187,188,189,190,191,192,193,194,195,196,197,198,199,200,201,202,203,204,205,206,207,208,209,210,211,212,213,214,215,216,217,218,219,220,221,222,223,224,225,226,227,228,229,230,231,232,233,234,235,236,237,238,239,240,241,242,243,244,245,246,247,248,249,250,251,252,253,254,255,256,257,258,259,260,261,262,263,264,265,266,267,268,269,270]. Bramley et al. [250] described a close relationship between EC_a_ readings from stony shallow soils and trunk circumference. However, sufficient predictors for vine vigour were not found in these investigations.

EM38 has more rarely been applied to apple orchards. Türker et al. [279] produced EC_a_ maps and compared them with yield and pomological characteristic maps. As a result, the highest value of a non-linear regression between EC_a_ and apple yield was determined with an *R*^2^ of 0.94.

### 5.2. Improvement of the Efficiency of Agricultural Field Experimentation

Only a few publications reported about the application of EC_a_ readings to improve the efficiency of field experiments. An accurate comparison of treatments within agricultural field experiments is the primary objective of these evaluations. Spatial soil variability can have adverse effects on the accuracy and efficiency of such trials (Table 8).

Kravchenko et al. [289] used EC_a_ as a covariate to improve the accuracy of P values on field with different levels of manure applications. Standard errors for the means of P with EC_a_ as a covariate were smaller than those for which EC_a_ was not used as a covariate. In soils with medium and high EC_a_ values, the control treatment (no manure) had a significantly lower P concentration.

Johnson et al. [204] applied field wide EC_a_ readings as a classification parameter for a block design. Blocks were located in homogeneous areas based upon measurements of soil parameters that are significant for yield. The authors noted that EC_a_ classification can be used as a basis for blocking only when EC_a_ and yield are correlated. On these sites, which were described by Johnson et al. [204], the dominating factors were salinity and clay content. The authors described the application of EC_a_ as a “compelling tool in statistical design”.

The initial point of the publication of Lawes and Bramley [288] is the fact that farmers and their advisers are often not able to implement methods that are necessary for evaluation trials on their farms. The authors explore a new and simple approach to the analysis of farmer strip trials and the spatial variability of treatment response. Yield data descriptions with a linear model that accounted for the spatial autocorrelation in the data and a moving pairwise comparison of treatments were applied by the authors. The results suggest that the pairwise comparison adequately identified treatment differences and their significance. This method can be readily implemented and expanded with EC_a_ readings, and it offers an important advance to facilitate on-farm experimentation using precision agriculture technologies.

Brevik et al. [173] indicated a need to investigate the application of EC_a_ techniques in fields with more homogenous soil properties. For these investigations, the authors selected a field with lacustrine-derived soils that exhibited only weak spatial variability in soil properties. The highly uniform EC_a_ readings obtained did not allow differentiation of soil map units with the EC_a_ data. However, the results did confirm the uniform nature of the soils in the field, a critical criterion for precision agriculture applications. An example of the application of conductivity values is given in Table 9 [4].

The relationships presented in Section 5.1 between EC_a_ and yield are here integrated in a variance of analysis (ANOVA) and an analysis of covariance (ANCOVA) with the target to model the multi-annual yield of the long-term experiment Dürnast 020. In the ANOVA only the factors “fertilizing level“ and “the form of fertilizer” have been considered. To enhance the accuracy of the simulation the covariates EC_a_ as well as topographical parameters have been added. The ANOVA procedure delivers with the fertilization level as the single influencing factor only a weak result (*R*^2^ = 0.185, RMSE = 3.26 dt ha^−1^). In contrast to this result the application of the ANCOVA introduced the factors fertilization level and fertilization no. and the covariate EC_a_ (EM38-h and EM38-v) in the simulation. The *R*^2^ of 0,875 and a RMSE with 1.29 dt ha^−1^ indicate a severe enhancement in comparison to the ANOVA. The partial eta-square illustrates that the introduction of the EC_a_ readings was the main reason of this improvement. The topographical parameter channelnet (channel network base level (-)) and TWI (topographical wetness index (-)) had only minor meaning.

Here, EC_a_ has been shown to be a useful indicator of soil variability. Compared to the standard analysis ANOVA, an ANCOVA with EC_a_ as covariate (and also topographical parameters) reduced RMSE and enhanced *R*^2^ for treatment means and improved the accuracy of this field experiment.

### 5.3. Additional Application of EM38 in Agriculture and Horticulture

Additionally, some publications describe the use of EC_a_ to assess environmental susceptibility and/or effects (Table 10).

Jaynes et al. [292] correlated EC_a_ readings with herbicide partition coefficients. The maps are useful for determining areas with a higher leaching potential for herbicide (atrazine) application. Olesen et al. [293] developed two different algorithms (an empirical model and a causal model) for spatially varying fungicide applications. Both models make use of a ratio vegetation index and EM38 measurements. EC_a_ maps describe the soil characteristics, in particular the soil clay content.

Hbirkou et al. [291] used EC_a_ maps for constructing relationships between ECa and the beet cyst nematode, *Heterodera schachtii*. This nematode prefers deep soil with medium to light soil and non-stagnic water conditions. Correlations between EC_a_ and nematode population density were moderate (*R*^2^ = 0.47) and strong (*R*^2^ = 0.74). Management maps based on EC_a_ and soil taxation maps indicated areas with different soil-related living conditions for H. schachtii. These maps could make farmers able to improve site-specific management strategies on nematode-infested fields.

Grigera et al. [131] created four EC_a_ zones from EC_a_ readings, based on ranges of both configurations using an unsupervised classification. Soil microbial groups were correlated with different soil C fractions in the upper soil (−15 cm) and were similar across EC_a_ zones. Zone distribution and biomarkers correlated in dependence of the fractions of particulate organic matter (fine particulate organic matter: bacterial (*R* = 0.85), actinomycetes (*R* = 0.71) biomarker concentrations; coarse particulate organic matter: bacteria *R* = 0.69, actinomycetes *R* = 0.48). In contrast, fungal (*R* = 0.77) and mycorrhizal (*R* = 0.48) biomarker concentrations were correlated only with coarse organic matter.

## 6. Application of EM38 in Archaeology

The application of the EM38 device is not restricted to soil properties; it also detects extrinsic components (Table 11).

Ferguson [298] applied EC_a_ values to find metal objects in a settlement area from the 18th century. Measurements of EC_a_ also appear to be suitable to search for graves [303]. Low values can indicate a proximity to metal, but high conductivity has been associated with grave shafts at one cemetery.

A more sophisticated procedure for archaeological detections was described by Dalan and Bevan [296]. An EM38 meter, which was operated in the inphase mode, measured the susceptibility of the top half-meter of soil. This susceptibility sounding was performed using a series of heights from 2 m to the surface, with readings taken at intervals of 5 cm. These measurements were analysed with the aid of the depth sensitivity function of McNeill [304]. In this manner, the authors could detect magnetic layers to a depth of 50 cm.

Viberg et al. [302] combined the EM38 with the MS2D (Bartington MS2 magnetic susceptibility meter). The anomalies contained in the survey data were explained by the subsequent archaeological excavation. A rubbish pit which consist mainly of organic material and fire-cracked stones was detected in both the MS2D and EM-38 data. This study of Simpson et al. [301] used additionally a fluxgate gradiometer measurements on an archaeological site. The results of the first survey showed very strong magnetic anomalies in the central field, which were caused by the brick remains of the castle. The most useful results with the EM38 were obtained from the magnetic susceptibility. Its anomalies corresponded well with the gradiometer anomalies. To enhance EC_a_ maps, Santos et al. [299] recommended a simple procedure to eliminate the effect of elevation on EC_a_. In the experience of the authors, soil anomalies are partly changed by changing the elevation within an investigation area according to the water table depth or the conductive sediment layer. With a linear dependence between conductivity and the site elevation the influence of topography was removed. Corrected EC_a_ maps substantially improved the recognition of anomalies. These maps also show a greater similarity with magnetic susceptibility maps, with both identifying archaeological structures of interest: a well-structured fireplace and a concentration of ceramic fragments.

## 7. Conclusions and Closing Remarks

There is no doubt that EM38 measurements have an increasing importance in exploration of areas, but weaknesses/unclarities of the method are also described in the literature:

- The interpretation and utility of EC_a_ readings are highly location and soil-specific; the soil properties contributing to EC_a_ measurements must be clearly understood. From the various calibration results, it appears that regression constants for relationships between EC_a_, EC_e_, soil texture, yield, etc. are not necessarily transferable from one region to another. Several factors affect the strength of the signal and therefore, the relationships. In addition to texture, salt concentration and other physicochemical properties, calibrations are further affected by the relative response of the signal according to depth, the non-linearity of the signal and the collinearity between horizontal and vertical readings. The soil parameter with the greatest influence on EC_a_ is also the best derivable.
- Only a few authors [108,196] account for the influence of the farming system, crop biomass, applications of fertilizer at the time of measurement on EC_a_ distributions. Most of the identified soil parameters that influence EC_a_ have significant interdependency and can thus provide multivariate effects on EC_a_.
- The modelling of EC_a_, soil properties, climate and yield are important for identifying the geographic extent to which specific applications of EC_a_ technology (e.g., EC_a_ – texture relationships) can be appropriately applied.
- In the case of detecting salinity, obviously better results are achieved if both EM38 readings (vertical and horizontal) are combined with EC_e_ values from different depth ranges. Nevertheless, Vlotman et al. [37] posed the question about the need for converting the EC_e_ from EC_a_. As McKenzie [24,25] showed, a classification of salinity tolerance level of different crops is also possible only with EM38 readings. A partitioning in areas of low, medium and high salinity with measurements in a single mode or with a combination of v- and h-mode is often a sufficient inventory of the salinity distribution. But it is necessary to take into account, that on the one field e.g., 60 mS m^−1^ has salt problems while another field with the same reading does not have such problems. Therefore EC_e_ will continue to be important at least in the near future.
- The quality of a regression is often determined by a sufficient range of dependent and independent variables. Delin and Söderström [124] noted that when the EC_a_ data were correlated with the clay content over the whole farm, the result was much better then when the correlation was restricted to single zones. This quality is also better if the target variable is also the dominant EC_a_-influencing factor.
- The construction of soil sampling designs with EC_a_ readings is limited to those properties that correlate with EC_a_. Other parameters require some other sampling approach such as random, grid, or stratified random sampling.

The world-wide application of the EM38 (and also of other soil sensors) is very varying:

- It seems that the detection of salinity is still the main area of application.
- Site-specific management in agriculture with the application of EC_a_ is still in Germany in an initial phase of adoption among farmers. Predicting the future is difficult. Nonetheless, a greater presence of site-specific crop management based on soil detection is to be hoped for.
- Furthermore in Germany increases the investigations in improving soil maps and in detecting soil functions, including: plant available water, sorption capacity, binding strength for heavy metals, filtering of unbound substances and natural soil fertility. Additionally, soil protection measures are also indicators for erosion prevention, retention of nutrients, and conservation/enhancement of carbon contents (based on good agricultural practice after Article 17, German Soil Protection Act). The selection of soil functions is based on the German Soil Protection Act (LABO—Bund-Länder-Arbeitsgemeinschaft Bodenschutz). Here it is not common sense to carry out this also with EM38. Until now it is not well known that, compared to traditional soil survey methods, EM38 readings can more effectively characterize diffuse soil boundaries and identify areas of similar soils within mapped soil units. This gives soil scientists greater confidence in their soil mapping.
- The application in forests is world-wide rather seldom. But also here is an enormous potential to improve the existing site maps and to test the water distribution between the trees.
- The improvement of evaluation of field experiments with EC_a_ readings as covariate is more rarely used. The spatial variability of soil properties can have adverse effects on the accuracy and efficiency of field experiments. Here is a great potential to take into account the soil conditions by using ECa readings.
- The fusion of the data of other sensors also shows great potential. The idea behind the combination of proximal soil sensors is that the accuracy of a single sensor is often not sufficient. The reading of one sensor is affected by more than one soil property of interest. The fusion of sensor data can overcome this weakness by extracting complementary information from multiple sensors or sources. Until now to an increasing extent, the readings of EM38 are evaluated in combination mainly with VIS–NIR and a gamma-ray-spectrometer.
- Many of the instruments measure at the point or sample scale, such as soil moisture probes and tensiometers, while remote sensing devices determine regional patterns. But these techniques are limited in the depth of penetration into the subsurface.

Here geophysical methods have a positive impact, obtaining data at a range of spatial scales across fields. This survey has shown that considerable progress has been made in detection and understanding of soil functions within the last decades. Applications of practical sensors such as the EM38 are needed to achieve sustainable agriculture, to optimize economic return and to protect the environment, especially the soil.

## Figures and Tables

**Table 1:** Overview with literature of relationships between EM38-EC_a_ and salinity.

Study	Parameters	Location of Investigation
Derivation of salinity with EC_a_ and EC_e_		
[8]	EC_a_ and EC_e_ relationships: classifying salt affected area	California, USA
[9]	Descriptions and formulations of EC_e_ and EC_a_; mathematical coefficients;	South Australia
[10,11]	Descriptions and formulations of EC_e_ and EC_a_; inverted salinity profiles;	South California, USA
[12]	EC_a_ and EC_saturated extract_, Na, Cl, Salinity maps with relation to yield Barley)	North-east Australia
[13]	Calibration EC_e_ and EC_av_, EC_ah_	Missouri, USA
[14]	EC_a_ and EC_1:5_ relationships to perform growth of Australian tree species on saline sites	Queensland, Australia
[15]	Formulations of EC_e_ and EC_a_	Egypt
[16]	Relationship EC_a_ and EC_e_, EC_a_ observations on establishing and growth of perennial pasture species	Australia
[17]	Salinity contour maps with EC_e_ and EC_av_, EC_ah_	Nnortheast Spain
[18]	Salinity classification system based on EC_1:5_ with groups of degrades	Henan, China
[19]	Formulations of EC_e_ and EC_a_	California, USA
[20]	EC_a_, EC_e_ to apply site specific management tech. on saline sites	California, USA
[21]	EC_e_ and EC_av_, EC_ah_ advanced calibrations reduce soil sampling from 200-300 to 36,	California, USA
[5,22]	Site calibration EC_e_ and EC_av_, EC_ah_	Saskatchewan, Canada
[23,24,25,26]	Formulations of EC_a_ and EC_e_; Salt tolerance of trees, forages, crops and turf grasses; survival and growth of eucalyptus and pastures in saline soils.	Alberta, Canada
[27]	Exchangeable sodium percentage and EC_e_ in relation to EC_a_	Illinois, USA
[28]	Soil survey with salinity regions; relationship EC_e_ and EC_a_ to detect salinity of irrigated districts	Aragon, Spain
[29]	Ranges of EC_a_ as classification system of saline areas	Victoria, Australia
[30]	Salinity classification system based on ranges of total dissolved salt concentrations, EC_1:5_ with groups of crops with different tolerances to rootzone salinity	Victoria, Australia
[31,32,33,34]	Descriptions and formulations of EC_a_, EC_e_, EC_p_ and EC ratios; multiple regression coefficients;	California, USA
[35]	Relationships of EC_e_ and EC_a,_ Soil salinity maps of different depth intervals and salinity profile maps at upstream and downstream of the field borders	Yazd Province, Iran
[36]	Monitoring spill of liquid manure occurred a few years ago	Manitoba, Canada
[37]	Formulations of EC_e_ and EC_a_ (India)	India (different regions)
[38,39]	Descriptions and formulations of EC_e_ and EC_a_; modeled coefficients;	NSW, Australia
[40]	Comparison EC_1:5_ - EC_e_ and EC_a_ to detect salinity in an early stage	Nakhon Ratchasima, Thailand
[41]	Comparison EC_e_ and EC_a_ to detect salinity	New Mexico, USA
[42,43,44,45]	Determination EC_e_ profiles with EC_a_ (EM38 and EM31); geostatistical methods to predict salinity from EC_a_ (EM38 and EM31), comparison calibration approaches;	NSW, Queensland, Australia
[46,47]	Ratio (EM38/EM31) sampling points to determine deep drainage and leaching fraction, EC_a_ and EC_e_; EC_a_ and clay; EC_a_ and deep drainage;	NSW, Australia
[48]	EC_e_, water content and EC_ah_, combined with cokriging	California, USA
[49]	Descriptions, formulations, classifications of EC_a_, EC_e_, EC_p_ and EC ratios	–
[50]	Overview salinity and determination	–
[51,52,53]	Detection subsurface saline material	Victoria, Australia
[54]	Calibration models EC_e_ and EC_a_ and water content over regional scale	Colorado, USA
[55]	Descriptions and formulations of EC_e_ and EC_a_, simple depth weighted coefficients;	North Dakota, USA
[56]	Depthwise calibration models EC_av_, EC_ah_ and EC_e_ and EC_1:5_ to construct inverted salinity profiles	Jiangsu, China
[57]	Comparison saturated paste and 1:1 soil to water extracts	Oklahoma, Texas, USA
[58]	Formulations of EC_e_ and EC_a_	Pakistan
[59]	Site calibration EC_e_ and EC_av_, EC_ah_	Navarre, Spain
[60]	Site calibration EC_e_ and EC_av_, EC_ah_	North Dakota, USA
[61]	Site calibration EC_(1:5)_ and EC_ah_	West Australia
[62]	Salinity calibration model to simulate EC_e_ from EC_a_	California, Minnesota, USA
[57]	Comparison saturated paste and 1:1 soil to water extracts	Oklahoma, Texas, USA
Construction of salinity maps		
[63]	Interpolation methods of EC_a_; EC_a_ maps as base for salinity maps/EC_e_)	Uzbekistan
[64]	Relation ECa-topography-salinity extension	Senegal
[65]	EC_a_-salinity areas	SE Australia
[66]	Salinity maps with stepwise data processing	Victoria, Australia
[67]	Mapping salinity with EM38, EM31 and Wenner array	Alberta, Canada
[68]	Geostatistical analysis of soil salinity data	–––––––
[69]	Salinity distribution within a field and combination with iodine tracer study	Cape Province, South Africa
[70]	Soil salinity maps with EC_a_, in relation to land use and soil/geology	South Australia
[71]	EC_a_ and visual agronomic survey of salinity	Punjab, Pakistan
[72]	Mapping of salinity plume in a sandy aquifer	North Dakota, USA
[73]	Detecting salt stores and evaluation of the risk of salinisation	NSW, Australia
[74]	EC_a_ maps by inverting data collected at various heights in the EM4SOIL software	Yazd Province, Iran
[75]	Salinity characteristics with PCA	California, USA
[76]	Comparison of multiple linear regression and cokriging	California, USA
[77]	Temporal changes in salinity using EC_a_	Aragon, Spain
[78,79,80]	Saline seep mapping and remediation; comparison salinity (EC_e_) and EC_a_ of different conductivity tools; saline seep mechanism in combination with hydrological modeling	Kansas, USA
[81]	Comparison salinity (EC_a_) between different land use	Australia
[82]	EM38 field wise	NSW, Australia
Salinity and field management		
[83]	Assessment of salinity by farmers	Australia
[84]	Effect of salinity on eucalyptus trees	SE Australia
[85]	Soil salinity and groundwater properties	Tunisia
[86]	Extension of groundwater acidity	NSW, Australia
[87]	EM38 and TDR: comparison of measuring methods	-
[88]	Assessment of soil quality properties with EC_a_	California, USA
[89]	ECa distribution in the landscape and as a consequence of evapotranspiration and phreatic rise	South Australia
[90]	Salinity in vineyards	Australia
[91]	EC_a_–salinity–water content	California, USA
[92]	Salinity management in cotton fields	California, USA
EM38 in combination with other sensors		
[93]	Comparison tools and methods detection salinity	Australia
[94]	EM38 in combination with satellite-based navigation methods	Alberta, Canada
[95]	Increasing precision of salinity with EM38 and EM31 (both EC_ah_) at various layers	Yellow River Delta, China
[96]	Hyperspectral data related to different soil salinization extent was combined with ECa order to establish a soil salinization monitoring model	Weigan River, China

**Table 2:** References indicating relationships between EM38-EC_a_ and soil texture.

Study	Texture	Texture Content (%)	EC_a_ (mS m^−1^)	*R*^2^		Location of Investigations
Europe						
[103]	Clay Silt Silt + Clay	not described	EC_av_: 10–110	0.28/0.53 * 0.14/0.49 * 0.25/0.71 * * with extracting TWI-trend		Wulfen, Kassow, East Germany
[116]	Clay Silt	4–16 7–36	EC_av_: 3–30	EC_av_: 0.55 (clay) EC_av_: 0.67 (clay + silt) (after factor scoring)		Brandenburg, Berlin, Germany
[120]	Clay	2–60	EC_av_: mean 13–92	EC_av_: 0.56		Saxony-Anhalt, Germany
[121]	Clay	2–45	EC_av_: 2–80	EC_a_: 0.66 EC_a_ corr: 0.85, corrected across field boundaries with neighbors regression		Bavaria, Germany
[122]	Clay	6–42	EC_av_, EC_ah_: 6–36	EC_av_: 0.08–0.38 EC_ah_: 0.13–0.33		Scheyern, Germany
[123]	Clay	7––32	EC_av_: 8–44 EC_ah_: 6-41	EC_av_: 0.21–0.44 EC_ah_: 0.13–0.67		Scheyern, Germany
	Silt	4–53	EC_av_: 8-44 EC_ah_: 6–41	EC_av_: 0.11–0.46 EC_ah_: 0.01–0.60		
	Sand	28–79	EC_av_: 8–44 EC_ah_: 6–41	EC_av_: 0.04–0.38 EC_ah_: 0.13-0.69		
[109]	Clay Silt Sand	2–25 5–69 5–50	EC_av_: 5–65	EC_av_: 0.76–0.76 EC_av_: 0.65–0.71 EC_av_: 0.00–0.69		3 fields around Bonn, Germany
[108]	Clay	3–48	EC_av_: 2–99 EC_ah_: 5–77	EC_av_: 0.76 EC_ah_: 0.74		South Germany
	Silt	4–71	EC_av_: 2–99 EC_ah_: 5–77	EC_av_: 0.67 EC_ah_: 0.67		
	Sand + gravel	15–67	EC_av_: 2–99 EC_ah_: 5–77	EC_av_: 0.76 EC_ah_: 0.74		
[124]	Clay	5–30	EC_av_: 9 (mean)	EC_av_: 0.94		Southwest Sweden
[125]	Clay	9–24	EC_av_: 4 EC_ah_: 32.2 approximate values two depths: 0–25 cm, 25–60 cm and 2 fields	EC_av_: 0.19–0.41 EC_ah_: 0.32–0.45		South Norway
	Silt	28–49		EC_av_: 0.006–0.52 EC_ah_: 0.002–0.56		
	Sand	33–61		EC_av_: 0.01–0.4 EC_ah_: 0.02–0.44		
	Gravel	3–11		EC_av_: 0.05–0.94 EC_ah_: 0.08–0.94		
[126]	Clay	about 5–40	EC_ah_: 6–26	EC_ah_: 0.63		South Norway
[127]	Clay Sand	23–44 39–67	EC_av_: 0–50	EC_av_: 0.55 EC_av_: 0.41		Moravia, Czech Republic
[128]	Clay	4–24		EC_av_: 0.49–0.67 (different dates on the same field)		Jütland, Denmark
[129]	Clay	2–56	EC_av_: 9–106 EC_ah_: 5–97	EC_av_: 0.81		East-Flanders, Belgium
[104]	Clay	topsoil: 14–24 subsoil: 3–27	EC_av_: 18–47 EC_av_: 12–36	(EC_av_* EC_ah_)^0.5^: 0.69 subsoil (EC_av_* EC_ah_)^0.5^: 0.16 topsoil		Flanders, Belgium
North America						
[106]	Clay	10–46 (mean values)	EC_av_: 1–54 EC_ah_: 1–56	EC_av_–30 cm: about 0.5 EC_ah_–30 cm: 0.3–0.56		North Carolina, USA
	Silt	20–35 (mean values)	EC_av_: 1–54 EC_ah_: 1–56	EC_av_–30 cm: 0.4–0.6 EC_ah_–30 cm: −0.3–0.56		
	Sand	40–70 (mean values)	EC_av_: 1–54 EC_ah_: 1–56	EC_av_–30 cm: about 0.4 EC_ah_–30 cm: −0.3–−0.6		
[130]	Clay Silt Sand	24–44 26–51 8–50	EC_av_ , EC_ah_: about 40, salinity affected	0.08 0.18 0.14	ln of geometric mean of EC_av_ and EC_ah_	California, USA
[112]	Clay	3–48	about EC_av,_ EC_ah_ 10-65	EC_av_: 0.11. EC_ah_: 0.08		Western California, USA
[131]	Clay	14–29	EC_av_: 19–35 EC_ah_: 14–26	EC_av_: 0.69 EC_ah_: 0.66		Nebraska, USA
[118]	Clay	12–32	EC_av_: 19–118	0.76		12 sites in Texas, USA
[132]	Clay	13–63	EC_av_: 30–65 EC_ah_: 38–83	EC_av_–30 cm: 0.55 EC_ah_–30 cm: 0.55		Central Missouri, USA
	Silt	33–81	EC_av_: 30–65 EC_ah_: 38–83	EC_av_–30 cm: 0.55 EC_ah_–30 cm: 0.55		
	Sand	6–11	EC_av_: 30–65 EC_ah_: 38–83	EC_av_–30 cm: 0.27 EC_ah_–30 cm: 0.27		
[3]	Clay Silt	13–36 31–67	EC_av_: 7–37	EC_av_: 0.55 EC_av_: 0.15 and 0.48 (2 fields)		North-central states, USA
[133]	Clay Silt Sand	about 5–40 unknown unknown	EC_av_:about 5–60	EC_av_: 0.36–0.77 EC_av_: 0.27–0.71 EC_av_: 0.21–0.36		Midwest USA
[100]	Clay Sand	10–32 52–85	EC_av_: 84.8 EC_ah_: 40.1	EC_ah_: 0.76 EC_ah_: 0.74		Southwest USA
Australasia						
[42,45]	Clay	about 30–85	EC_av_:80–200 (salt affected)	EC_av_ 0.62 and 0.64		NSW, Australia
[134]	Clay	about 40–65	EC_av_:30–210	EC_av_: 0.72		NSW, Australia
[119]	Clay	15–58	EC_av_: 5–159 EC_ah_: 13–147	EC_av_: 0.66 EC_ah_: 0.67 combination of EM34 and EM38 in different modes:0.79		NSW, Australia
[135]	Clay	about 20–45	about 10–36	EC_av_: 0.72 EC_ah_: 0.65		Manavata, New Zealand
Asia						
[136]	Clay Silt Sand	1.5–41.3 6.5–33.5 45.8–91.0	EC_av_: 1–40	topsoil: 0.47 (on average)		Sri Lanka
Unknown						
[137]	Clay	12–20	EC_av_: 7–20 EC_ah_: 7–15	EC_av_: 0.78 EC_ah_: 0.80		Not described

**Table 3:** Literature describing relationships between EM38-EC_a_ and parameters of soil water.

Study	Parameters	Location of Investigations
Water content		
[138]	Water content	Iowa, USA
[139]	Water content	Iowa, USA
[112]	Water content	South California, USA
[91]	Water content	California, USA
[140]	Water content, water table depth	New Zealand
[141,142]	Water content	Ontario, Canada
[143]	Water storage [mm]	Minnesota, USA
[144]	Soil drainage classes	Illinois, USA
[145]	Soil water content (θ_v_, θ_w_), ±3%	South Dakota, USA
[146]	Plant available water content	Missouri, USA
[147]	Water content	Columbia County, USA
[148,149]	Volumetric water content	Texas, USA
[122]	Water content: EC_av_: 0.39; EC_ah_: 0.26 Plant available water content: EC_av_: 0.31; EC_ah_: 0.29	Bavaria, Germany
[123]	Water content EC_av_: 0.04–0.26; EC_ah_: 0.16–0.64	Bavaria, Germany
[150]	Water content	Florida, USA
[3]	Water content	North-central USA
[151]	Water content with EM38 and ASD spectrometer	Quebec, Canada
[102]	Repeated EC_a_ measurements for determining water content	Pennsylvania, USA
[152]	Detection of available water content from EC_a_, for using in the yield software ADSIM	WA, Australia
[153]	Repeated EC_a_ measurements and relation to water content (irrigation)	Queensland, Australia
[115]	Available water content and soil water deficit from texture finess classes and EC_a_	Cambridgeshire, UK
[154]	EC_a_ in combination with GPR to predict field wide water content	South-east Italy
[155]	Soil water content, soil bulk density	South Dakota, USA
Groundwater, water table depth, water drainage		
[156]	Water table depth using geophysical and relief variables	Darling River, Australia
[9]	Groundwater recharge	South Australia
[157]	Depth to groundwater table	Montana, USA
[158]	Soil drainage classes	Iowa, USA
[159]	Characterizing of water and solute distributions in the vadose zone with readings of EM38 and borehole conductivity meter	New Mexico, USA
[160]	Water table depth	Florida, USA
[161,162]	Detection of areas with different water movements	Tennessee, USA
[46]	Deep drainage risk	Australia
[163]	Hydraulic conductivity of palaeochannel in alluvial plains	NSW, Australia
[42,45]	Deep drainage (mm/year) with a 4-parameter broken-stick model fitted to EC_av_ beyond 120 cm	Australia
Irrigation		
[164]	Irrigation effectiveness/drainage	California, US,
[165]	EC_a_ – soil available water holding capacity on two variable-rate irrigation scenarios	New Zealand
[166]	EC_a_ for quick assessment of deep drainage under irrigated conditions in the field.	Australia

**Table 4:** Literature indicating derivations of soil types and patterns as well as further soil parameters from EM38-EC_a_.

Study	Investigation Object	Location of Investigation
Soil types		
[171]	Separation between Natraqualf and Ochraqualf	Tennessee, USA
[172]	Soil types, yield maps	Virginia, USA
[173]	EC_a_ to derive more homogeneous lacustrine-derived soils	Iowa, USA
[174]	Soil pattern as basis of management zones	England
[175]	Soil boundaries	Denmark
[158]	Soil map unit boundaries, detection of inclusions	Iowa, USA
[2]	Refine and improvement of soil maps	-
[176]	Soil types with clusteranalysis	Elbe-Weser-region, Germany
[177]	Detection of areas with sulfidic sediments and coastal acid sulfate soils	NSW, Australia
[128]	Soil types	Jütland, Denmark
[178]	Soil boundaries between clay loam and sandy loam soils	Cambridge, UK
[179]	Soil types, in combination with terrain parameters and other sensors	NW Victoria, Australia
[102]	Repeated EC_a_ measurements for determining soil types	Pennsylvania, USA
[180]	Inversion of EM38 and EM34 sigma-a data to detect the areal distribution of soil types	Darling River, Australia
[181]	Distinguishing between soils with cambic pedogenic horizons and argillic horizons; boundaries of soil map units	Texas, USA
[182]	Supporting delineation of spatial distribution of C content	Harz region, Germany
Soil depth to horizons/layers/discontinuities/borders		
[183]	Depth to limestone bedrock and clayey residuum	Florida, Pennsylvania, USA
[184]	Depth of claypan soils	Missouri, USA
[185]	Soil depth sounding	East, south Germany
[5]	Soil depth sounding	Ontario, Canada
[186]	Depth to sand and gravel	Unknown
[187]	Depth of sand deposition	Missouri, USA
[188]	Layer depth, EC_a_ as auxiliary variable	North Netherlands
[189]	Depth of the Tertiary substratum	Flanders, Belgium
[190]	Soil depth to petrocalcic horizon	Utah, USA
[191]	Soil depth to bedrock (loess above basalt)	Idaho, USA
[192]	Bulk density and EC_a_	Iowa, USA
[193]	Boulder clay depth	North Netherlands
[194]	Linear, negative relation between EC_a_ and topsoil layer thickness	Fuxin, China
[195]	Bayesian method to map the clay content of the B_t_ horizon associated with the control of encroaching trees	South Africa
[1,196,197,198]	Depth to claypan soils	Missouri, USA
Further soil properties		
[88]	Soil properties and cotton yield	California, USA
[199]	Soil properties and cotton yield	California, USA
[112]	Water content, cation exchange capacity, cations and anions in saturation extract and exchangeable, B, Mo, pH, C, N,	West California, USA
[132]	Cation exchange capacity, C, N, P, soil enzyme, microbial biomass, hydr. Sat. K., bulk density	Missouri, USA
[3]	Water content, cation exchange capacity	North-central states, USA
[45]	CEC in salt affected soils	NSW, Australia
[200]	CEC in dependence of EM38, EM31, 3 remotely sensed (Red, Green and Blue spectral brightness), 2 trend surface (Easting and Northing) variables	NSW, Australia
[201]	Exchangeable Ca, Mg, cation exchange capacity	Ontario, Canada
[124]	EC_a_ as a covariable in cokriging improved the prediction of pH, clay, SOM	Sweden
[202]	EC_a_ in relation to water content, yield, CEC, clay silt, organic matter	Brandenburg, Saxony-Anhalt, Germany
[131]	C, total dissolved solids, depth of topsoil	Nebraska, USA
[203]	Soil organic carbon and classifing with fields normalized EC_a_	Andalucia, Spain
[204]	N-dymanics for management zones	Nebraska, USA
[176]	Precision agriculture: combination of EC_a_ and soil parameters (clay, yield, plant available water)	Mecklenburg, Germany
[205,206]	Compaction in paddy rice fields by puddling	Bangladesh
[207]	EC_a_ as subsidiary variable for interpolation	Missouri, USA
[208]	Soil compaction	Silsoe, UK
[209]	Relations leaching rates to EC_a_	NSW, Australia
[210]	EC_a_ as subsidiary variable for interpolation of P, K, pH, organic matter and water content	Iowa, USA
[211]	Simple linear inversion of EC_a_ to simulate magnetic susceptibility	-

**Table 5:** Literature describing selection of areas for soil sampling with EM38-EC_a_.

Study	Investigation Object	Location of Investigation
[59]	Soil sampling points	Ebro River, Spain
[199]	Sampling design	West California, USA
[237]	EC_a_ base sampling design: response surface sampling design (RSSD), stratified random sampling design (SRSD)	California, USA
[228]	Soil sampling design pH	NSW, Australi,
[238]	Mapping sodium affected soils	Great Plains, USA
[204,239]	Soil sampling design, soil units	West California, USA
[100,236,240,241]	Soil sampling design	Southwest USA
[115]	Sampling design for loacation of neutron probe access tubes	Cambridgeshire, UK
[242]	VQT method (variance quad-tree) in combination of relief data and EC_a_	Jiangsu Province, China,
[235]	Optimum locations for soil investigations	Brandenburg, Germany

**Table 6:** Composition of literature with derivations of yield maps, management zones and selection of areas for fertilization with EM38-EC_a_.

Study	Investigation Object	Location of Investigation
[172]	Yield maps, Soil types and EC_a_	Virginia, USA
[106]	EC_a_, NIR, elevation, slope with k-means clustering to define management zones	North Carolina, USA
[65]	Help for define management options with EC_a_	SW, Australia
[250]	Development of predictors of vine yield from EC_a_	New Zealand
[251]	Management zones in viniculture	Clare Valley, Australia
[103]	Relationship EC_a_ crop yield	North, east Germany
[252]	Management zones on soil NO_3_ and P sampling variability	South Dakota, USA
[253,254]	N-management zones	Belgium
[130,199,255]	Soil properties and cotton yield	California, USA
[174]	Soil pattern as basis of management zones	England
[12]	Identifiing management classes with ECa (measured at high and low water content)	North-east Australia
[154]	Multi-sensor data (EM38, GPR, FieldSpec) to delineate homogeneous zones	Italy
[256]	Relationships EC_a_, N-fertilizing demand	Southwest Sweden
[257]	Relationship EC_a_ crop yield , management zones	Brandenburg, Germany
[258]	Establishing of management zones with Corg, clay, NO_3_, K, Zn, EC_a_, corn yield data	Colorado, USA
[259]	Correlations ECa with yield, sugar content, piercing force, Kramer energy in a single year	Peleponnese, Greece
[260]	Relationship EC_a_ crop yield, management zones	Missouri, USA
[261]	Management zones and N applications	Missouri, USA
[262]	Management zones delineation software	Missouri, USA
[224]	EC_a_ to predict NO_3_-concentration	Dakota, USA
[131]	EC_a_ zones	Nebraska, USA
[263]	Distribution of legumes in pastures in dependence of EC_a_ and slope	Iowa, USA
[176]	Soil types (derived from EC_a_) related to yield, K, Mg	Elbe-Weser-region, Germany
[92]	Management zones salt affected sites	California, USA
[264]	Development of key properties for delineation management zones	North Belgium
[265]	Management zones in a paddy rice field with EC_a_	Bangladesh
[226,266]	Relationship EC_a_ crop yield	Iowa, USA
[267]	Management zones with yield, elevation and EC_a_	Iowa, USA
[132]	Relationship EC_a_ crop yield	Missouri, USA
[268]	ECa-maps to derive management zones	Iowa, USA
[269]	Relationship EC_a_ crop yield, terrain attributes	Iowa, USA
[213]	Relationship and classification EC_a_ crop yield	North central Missouri, USA
[270]	Managing and monitoring variability in vineyards	Australia
[271]	Management zones with yield, elevation, EC_a_, aerial photos	Nebraska, USA
[272]	Site-specific management of grassland	Ireland
[249]	Comparison EC_a_ – German national soil inventory (Bodenzahlen)	Bavaria, Germany
[273]	Lime applicationto reduce subsoil acidity	Western Australia
[225]	Relationships EC_a_, N-fertilizing zones	Saxonia, Germany
[274]	Senor application in viticulture	Australia
[275]	Multiyear EC_a_ – yield relationship	Victoria, Australia
[276]	Delineation of site-specific management-zones with ECa and topographic parameters	Nile Delta, Egypt
[277]	Data fusion (Terrian attributes, EC_a_, yield, aerial imagers)	Minnesota, USA
[179]	Yield zones, yield per year, in combination with terrain parameters and other sensors	North West Victoria, Australia
[164]	Relationship EC_a_ crop yield	Bavaria, Germany
[196]	Relationship EC_a_ crop yield	Missouri, USA
[278]	Relationship ECa − volumetric water content (−35 cm) – yield	NRW, Germany
[279]	EC_a_ and yield of apples	Ankara, Turkey,
[42,45]	Sampling points with ratio (EC_av_-EM38/EC_a_-EM31)	NSW, Australia
[245]	Management zones and multilevel sampling scheme	Central Iowa, USA
[280]	Management zones with EC_a_ relative differences (ϑ_ij_ , Eq. 31)	SW Spain
[104]	Management zones (delineated mainly with subsoil clay from ((EC_av_* EC_ah_)^.5^)) delivered from EC_a_)	Flanders, Belgium
[281]	Characterization of soil variation by key variables: pH, EC_a_, organic matter	Flanders, Belgium
[121]	Interpolation of EC_a_ across field boundaries	Bavaria, Germany
[282]	EC and soil inorganic N (no EM38-EC_a_)	Nebraska, USA

**Table 7:** Regressions between EC_a_ and multi-annual mean of yield (wheat) of the long-term experiment Dürnast 020 in dependence of fertilization level (see Figure 1).

Yield (dt ha^−1^)	Configuration	N	Equation	*R*^2^ Significance
Control plots	Vertical	12	101.33 − 1.411 × EC_a_	0.67 ***
	Horizontal	12	64.61 − 0.758 × EC_a_	0.81 ***
Fertilized plots (low)	Vertical	42	106.85 − 0.81 × EC_a_	0.36 **
	Horizontal	42	53.466 + 1.394 × EC_a_ − 0.025 × EC_a_^2^	0.76 ***
Fertilized plots (high)	Vertical	42	111.2 − 0.811 × EC_a_	0.22 *
	Horizontal	42	76.853 + 0.361 × EC_a_ − 0.012 × EC_a_^2^	0.67 ***

n.s. > 0.05, * 0.05 ≥ *p* > 0.01, ** 0.01 ≥ *p* > 0.001, *** *p* ≤ 0.001.

**Table 8:** Applications of EM38-EC_a_ for improving the efficiency of field experiments.

Study	Investigation Object	Location of Investigation
[173]	EC_a_ to derive more homogeneous lacustrine-derived soils	Iowa, USA
[204]	Classification parameter for block design	California, USA
[287]	P-content in a field experiment with different levels of manure applications	Michigan, USA
[288]	Comparison of yield between strip trials, partly EC_a_; simplified evaluation method	South, west Australia

**Table 9:** Simulation of the yield (1980–2012) with ANOVA and ANCOVA with the factors fertilizing level and fertilizer-no. and the covariates ECa and relief parameters.

Target Variable, Years	Model and Effects	Significance	Partial Eta-Square	Adjusted *R*^2^	RMSE (dt ha^−1^)
Yield (dt ha ^−1^), mean 1980, 1983, 1986, 1989, 1992, 1995, 1998, 2001, 2004, 2007, 2010, 2012	Adjusted model Constant Fertilization level Fertilizer no. Fertilization level*Fertilizer no.	0.008 0.000 0.000 0.414 0.971	0.313 0.998 0.258 0.081 0.018	0.18	3.26
Yield (dt ha ^−1^)^3^, mean (1980, 1983, 1986, 1989, 1992, 1995, 1998, 2001, 2004, 2007, 2010, 2012	Adjusted model Constant Fertilization level Fertilizer no. Fertilization level* Fertilizer no. EC_a_ (EM38-h)^3 lg10(EC_a_ (EM38-v)) Channelnetwork^3 TWI^3	0.000 0.007 0.000 0.000 0.145 0.000 0.000 0.001 0.024	0.904 0.106 0.764 0.341 0.131 0.275 0.276 0.144 0.075	0.88	1.29

Significance: n.s. > 0.05, * 0.05 ≥ *p*>0.01, ** 0.01 ≥ *p* > 0.001, *** *p* ≤ 0.001; Partial eta-square: Measure of sensitivity to the correlated independent variables; Adjusted *R*^2:^ adjusted *R*^2^ (coefficient of determination).

**Table 10:** Additional applications of EM38-EC_a_ in agriculture and horticulture.

Study	Investigation Object	Location of Investigation
[234]	Corg, K, pH, Bray-2 P,	Louisiana, USA
[290]	Detecting soil properties as indicators for population density of Redheaded cockchafer (*Adoryphourus couloni*)	Victoria, Australia
[215,217,219]	Specific ions that are associated with animal waste	Nebraska, USA
[220]	N decomposition, organic and artificial fertilizer	Nebraska, USA
[221]	EC_a_ as an indicator of N gains and losses, available N sufficiency for corn in early stage and NO3-N surplus after harvest	Nebraska, USA
[291]	EC_a_ as indicator for soil conditions which are prefered by *Heterodera schachtii*	North Rhine-Westphalia, Germany
[292]	Herbicide partition coefficients	Iowa, USA
[233]	Variation in soil testing P	Missouri, Oklahoma, USA
[293]	Part of fungicide application models in combination with ratio vegetation index	Denmark
[294]	Weed distribution, herbicide injury in dependency of EC_a_	North Rhine-Westphalia, Germany
[222]	NH_4_, K in animal slurries	Ireland

**Table 11:** Additional applications of EM38-EC_a_ in archaeology.

Study	Investigation Object	Location of Investigation
[295]	Detection of graves with inphase and quadphase readings	Maryland, USA
[296,297]	Prehistoric earthworks with measurements in inphase mode	Ohio, USA
[298]	Metal objects from the 18th century	Canada
[299]	Removing of the effect of elevation on the distribution of EC_a_ readings	Santa Catarina State, Brazil
[300]	Comparison EM38 fluxgate gradiometer	Belgium
[301]	Medieval manor in the dutch polders	Netherlands
[302]	Area prospection with EM38 and MS2D	Tundra region, Sweden

## Abbreviations

CEC	Cation exchange capacity
EC_a_	Apparent electrical conductivity
EC_av_	Apparent electrical conductivity, measured in vertical mode
EC_ah_	Apparent electrical conductivity, measured in horizontal mode
EC_e_	Electrical conductivity of aqueous soil extracts EC_1:5_, EC_1:2_ or EC_1:1,_ soil/water suspensions)
EC_p_	EC_a_ calculated by using predictive equations
EC_ref_	Quotient of the measured EC_a_ and the EC
θ_v_, θ_w_	Weighted water content after vertical and horizontal mode
Z	Soil depth
